# Collective cell migration without proliferation: density determines cell velocity and wave velocity

**DOI:** 10.1098/rsos.172421

**Published:** 2018-05-02

**Authors:** Sham Tlili, Estelle Gauquelin, Brigitte Li, Olivier Cardoso, Benoît Ladoux, Hélène Delanoë-Ayari, François Graner

**Affiliations:** 1 Laboratoire Matière et Systèmes Complexes, Université Denis Diderot - Paris 7, CNRS UMR 7057, Condorcet building, 10 rue Alice Domon et Léonie Duquet, 75205 Paris Cedex 13, France; 2 Mechanobiology Institute, Department of Biological Sciences, National University of Singapore, 5A Engineering Drive, 1, 117411 Singapore; 3 Institut Jacques Monod, Université Denis Diderot - Paris 7, CNRS UMR 7592, Buffon building, 15 rue Hélène Brion, 75205 Paris Cedex 13, France; 4 Univ. Lyon, Université Claude Bernard Lyon 1, CNRS UMR 5306, Institut Lumière Matière, Campus LyonTech - La Doua, Kastler building, 10 rue Ada Byron, 69622 Villeurbanne Cedex, France

**Keywords:** cell monolayer, migration, instability, wave, strain, polarity

## Abstract

Collective cell migration contributes to embryogenesis, wound healing and tumour metastasis. Cell monolayer migration experiments help in understanding what determines the movement of cells far from the leading edge. Inhibiting cell proliferation limits cell density increase and prevents jamming; we observe long-duration migration and quantify space–time characteristics of the velocity profile over large length scales and time scales. Velocity waves propagate backwards and their frequency depends only on cell density at the moving front. Both cell average velocity and wave velocity increase linearly with the cell effective radius regardless of the distance to the front. Inhibiting lamellipodia decreases cell velocity while waves either disappear or have a lower frequency. Our model combines conservation laws, monolayer mechanical properties and a phenomenological coupling between strain and polarity: advancing cells pull on their followers, which then become polarized. With reasonable values of parameters, this model agrees with several of our experimental observations. Together, our experiments and model disantangle the respective contributions of active velocity and of proliferation in monolayer migration, explain how cells maintain their polarity far from the moving front, and highlight the importance of strain–polarity coupling and density in long-range information propagation.

## 1. Introduction

Collective migration of cells connected by cell–cell adhesion occurs across several time scales and length scales in numerous biological processes like embryogenesis (notably gastrulation), wound healing, regeneration or tumour metastasis [1–4]. To study such long-range information propagation mediated by mechanical stress in tissues, *in vitro* reconstructed assemblies of cohesive cells are useful experimental model systems [5,6] where each individual cell can grow, divide, die and migrate. In two-dimensional (2D) monolayers, cells interact with each other biochemically and mechanically, for instance through adhesion, and have a richer migration behaviour than single cells. It is possible to constrain geometrically and reproducibly control their collective migration. Patterned substrate of adhesive strips enable to investigate the tissue global response to active processes such as cell migration [5,7] or cell division [8], and quantitatively test the impact of drugs like blebbistatin [9]. Madin–Darby canine kidney (MDCK) cell monolayers enable comparisons of experiments, simulations and theories [10–15]; 2D images are easier to obtain and analyse than three-dimensional (3D) ones, especially to extract physical quantities such as cell velocity, density, shape and deformation [12,16].

When monolayers are grown on a substrate, the latter acts as a source of external friction on cells [5,7,11,17]. If it is deformable (made of soft gel or covered with pillars), it acts as a mechanical sensor for traction force microscopy to quantify forces exerted by cells on the substrate, which are the opposite of forces exerted by the substrate on the cells [18–20]. Beside these external forces, mechanical stresses within the monolayer arise from cell-level processes which include: cell-volume change [21] and division [8]; competition between the adhesion to the substrate, the intercellular adhesion and the cell contractility [22]; cryptic lamellipodia extending from one cell below its neighbours [23].

The emergence of large-scale polarized movements within epithelial cell monolayers largely depends on mechanical factors and external geometrical constraints [7,13,16,24]. Loza *et al.* [25] (using human breast epithelial cells) showed that cell density and contractility control transitions in collective shape, and could predict in vivo collective migration in a developing fruit fly epithelium. Microfluidic channel experiments have shown that the flow velocity of the front can be decomposed into a constant term of directed cell migration superimposed with a diffusion-like contribution that increases with density gradient [26]. In the context of a cell monolayer collectively spreading and invading a free space, highly motile leader cells can appear [27] and locally guide small organized cohorts of cells [10]. The cell velocity decreases with the distance to the moving front [11], while both the cell density and the stress increase with the distance to the moving front [5]. Bulk cellular motions also display large-scale coordinated movements of cell clusters that can be seen by the emergence of a typically 200 μm correlation length for the velocity field and large-scale polarization [9,28].

Serra-Picamal *et al.* [11], by confining cells on a strip then releasing the confinement, observed two periods of a mechanical wave, propagating backwards from each front, made visible by oscillations of the cell velocity and its gradient, and suggesting how stress mediates collective motion. Mechanical force propagation has been reported during the collision of two epithelial cell layers to explain the formation of tissue boundaries [29]. Similar wound healing experiments displayed a wave of coordinated migration, in which clusters of coordinated moving cells were formed away from the wound and disintegrated near the advancing front; this wave could be amplified by the hepatocyte growth factor/scatter factor [30]. Confluent epithelial cells confined within circular domains exhibit collective low-frequency radial displacement modes as well as stochastic global rotation reversals [31,32]. While oscillations at smaller scales are common in embryogenesis (cell size and minute period [33–36]) or myxobacteria swarms (a few cell sizes, 1–100 min period [37]), here in confluent monolayers the oscillation scale is that of a tissue size and of hours, reminiscent of somitogenesis (for review of models, see [38]).

Even though the appearance of cell coordination and waves in collective migration experiments is crucial to understanding development and associated pathologies, it remains poorly documented. Migration and division contributions to the front velocity are entangled. Moreover, cell number is constantly increasing due to cell division, which leads to jamming and slowing of the migration. This usually limits the experiment duration to a few hours. The experimental uncertainty limits the possibilities of quantitative comparisons with models. The process which determines the velocity direction and amplitude of a cell far from the migrating front is not fully understood. In particular, it is still not clear how cell migration is sensitive to the distance to the migrating front and how cells maintain their polarity far from the migrating front. To improve our understanding, distinguish between the models and constrain their parameters, varied and controlled experimental data are required.

Here, we significantly improve experimental reproducibility and signal-to-noise ratio, and provide a precise analysis (§2). We observe a coherent collective cell migration over several millimetres, quantify average cell velocity profile and waves that develop on top of it and identify the roles of density and lamellipodia (§3). Our minimal model of strain and polarity coupling suggests an interpretation of these experimental observations (§4). Finally, by discussing and comparing the experiments and the model, we quantitatively confirm several preliminary results found in the literature, and add new results and insights regarding the role of mechanics in collective cell migration (§5).

## 2. Material and methods

**Figure 1.**
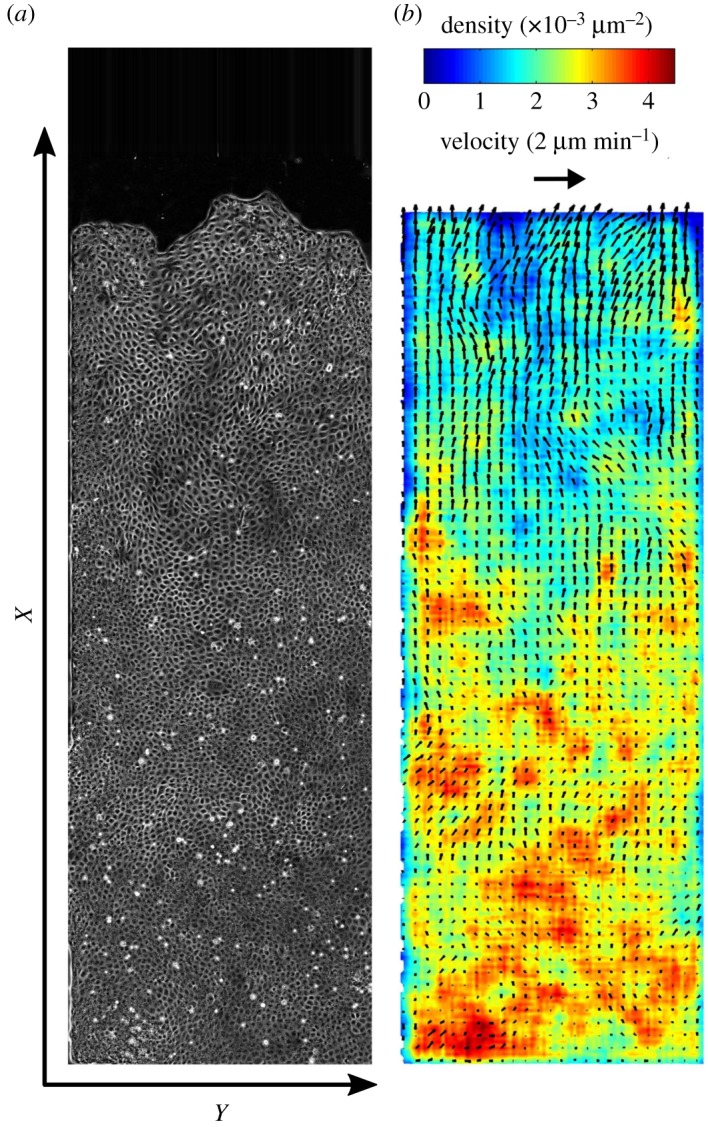
Cell migration. (*a*) A monolayer of MDCK cells, initially confined, is released. It expands (electronic supplementary material, movies S1–S3) along the adhesive strip towards empty space (direction of increasing *x*). Mitomycin C is added to inhibit divisions. Phase-contrast image of cell contours, taken at *t*=11 h 30 min (i.e. after approx. 16 h 30 min of migration). Strip total length 4 mm (most of it is visible here), width 1 mm. (*b*) Corresponding 2D fields of cell velocity and density. Scale arrow: $2\;\text{μ}m\;\min^{-1}$.

Section (a) explains how, by inhibiting cell division, we see a decrease in cell density due to migration. We observe a steady collective migration over a day or more, without reaching jamming densities. We focus on such long-distance migration and the strip length is adapted accordingly. Strips are narrow to prevent front shape instabilities, and the cell flow is essentially one-dimensional (1D) (figure 1; electronic supplementary material, figure S1 and movies S1–S6). Section (b) explains how we first average out the velocity field over several hours to characterize the mean cell velocity profile in the monolayer bulk. We then quantify the fluctuations around this average with an unprecedented signal-to-noise ratio using wavelet analysis.

### 2.1. Experiments

The micropattern of fibronectin is printed according to the following standard soft lithography technique, robust to small changes in the procedure. Patterned PDMS stamps prepared from silanized wafers are incubated for 45 min at 37°C or 60 min at room temperature with a solution of 50 μg ml^−1^ of fibronectin (Sigma) and 25 μg ml^−1^ of Cy3-conjugated fibronectin. A thin layer of rigid PDMS (10% of reticulating agent) is spin-coated on a 35 mm plastic Petri dish and cured for 2 h at 80°C or overnight at 65°C. The Petri dish is exposed to UV for approximately 20 min in order to activate the PDMS surface. After incubation, stamps are dried and pressed on the UV-activated PDMS surface in order to transfer fibronectin. A 2% Pluronic F-127 (Sigma) solution is added to the Petri dish to chemically block the regions outside of the fibronectin pattern for 1 h at room temperature. The Pluronic solution is removed after 1 h and the Petri dish is rinsed three to six times with a PBS solution.

We use the same MDCK strain as in [9]. A stable cell line was created using Histone GFP [39] using the DreamFect Gold Transfection Reagent (Oz Biosciences).

A batch has three to six strips, with the same initial MDCK cell density, lengths up to 4 mm. Strip widths range from 200 μm to 1 mm (at least equal to the typical 200 μm correlation length for the velocity field [9,28]) and do not affect the results presented here. Different batches correspond to different initial cell densities, tested at least twice each.

Suspended cells are deposited and allowed to attach for one to a few hours. Non-attached cells are rinsed, while attached cells grow and divide until full confluence. The confining PDMS block is removed. Some cells might detach, so the monolayer is rinsed again and left for a few hours. The monolayer starts to migrate along the whole accessible strip, expanding towards the empty surface where cells adhere to fibronectin, and not towards outside regions chemically blocked using Pluronic.

**Figure 2.**
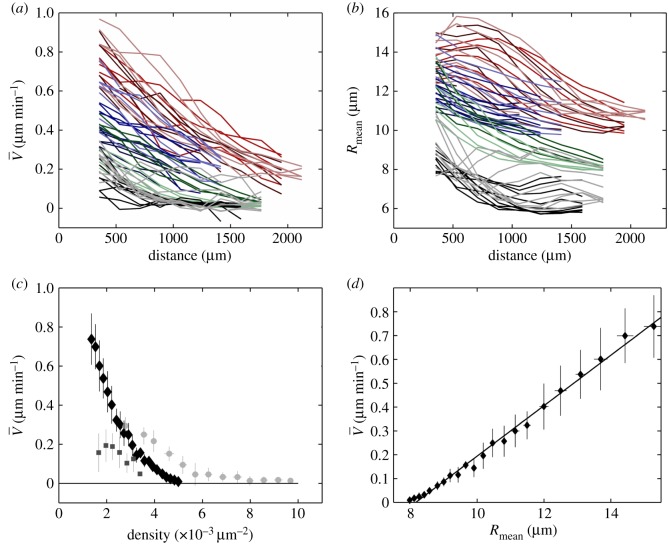
Cell velocity and density profiles. (*a*,*b*) Large-scale profiles. Large-scale average of (*a*) cell velocity $\bar{V}$ and (*b*) mean effective cell radius $R_{\mathrm{mean}}={\left(\pi \bar{\rho }\right)}^{-1/2}$, plotted versus distance *d* to the moving front (oriented from the front towards the cell reservoir). Each colour marks a different batch, with initial cell radius (from reservoir to front): red, 10–12.5 μm; blue, 10–11 μm; green, 8–10.5 μm. For a given colour (i.e. batch), each shade marks a different strip. For a given shade (i.e. strip), each data point is the average in a 176 μm×180 min bin. Grey and black curves are the same, for control experiments without mitomycin C, with initial density (from reservoir to front): grey, 8–10 μm; black, 7.7–7.9 μm. (*c*,*d*) Cell velocity–cell radius correlation. Same data as in (*a*,*b*), with mitomycin, binned and plotted as $\bar{V}$ versus cell density $\bar{\rho }$ (black diamonds) (*c*) or versus *R*_mean_ (*d*), with a linear fit $\bar{V}=0.106\;R_{\mathrm{mean}}-0.864$ (*R*=0.9931); *N*=8 strips. Light grey circles: control experiments without mitomycin C; *N*=3 strips. Grey squares: experiment with the drug CK666 to inhibit lamellipodia; *N*=5 strips. Horizontal and vertical bars: standard deviation (s.d.) within each bin.

To vary the initial cell density, we vary the amount of deposited cells and/or the time they are left to proliferate; we always begin with confluent monolayers. We do not measure cell volumes; we expect they are all similar at the time of deposition and that the main contribution to their variation is that of cell cycles. When the imaging begins and the density is measured, the monolayer has already migrated for a few hours (see below), so the initial density varies spatially from the reservoir to the front (for details, see caption of figure 2*a*,*b*).

To decrease the division rate, 8 μl of a 0.5 mg ml^−1^ mitomycin C solution (aliquoted, stored at −20°C and used within a day after thawing) is added to 1 ml cell culture medium, and cells are incubated at 37°C for 1 h [31,40]. They are then abundantly rinsed with fresh 37°C medium to prevent the toxicity effects reported for 12 h exposure to mitomycin [28]. After 3 h the division rate is less than a fifth of the initial one (electronic supplementary material, figure S2), and the rate of extrusions also strongly decreases. Control experiments are performed in standard conditions, with proliferating cells (no mitomycin C added).

To test the role of lamellipodia, we prepare a 100 μM solution in DMSO of CK666, namely 2-Fluoro-*N*-[2-(2-methyl-1H-indol-3-yl)ethyl]benzamide, a selective inhibitor of actin assembly mediated by actin-related protein Arp2/3 (IC50=17 μM) [41–43]. Aliquots are stored at −20°C and used within two weeks of preparation. The solution is added to the cells after approx. 1 day of migration and is not rinsed. Lamellipodia (both cryptic and front ones) are no longer detectable (electronic supplementary material, movie S7).

Two hours after having added the mitomycin, we take the first image of the movie and define it as *t*=0. Live imaging of monolayers is performed in the Nikon BioStation IM, a compact cell incubator and monitoring system, with an air objective (CFI Plan Fluor 10X, Nikon). Phase-contrast and fluorescent imaging are used to observe, respectively, cell contours and cell nuclei. The interframe time interval is 5 min for 1 mm wide strips and 6 min for 200 μm wide strips.

Dead or extruded cells appear as bright spots which can be removed by manual image intensity thresholding. The contrast is adjusted separately on each colour channel, and a blur with 2 pixel radius removes sharp intensity fluctuations. To obtain the whole view of the confined monolayer, up to 6 (for 2003 μm wide strips) or 20 (for 1 mm wide strips) microscope fields of view are merged using the Grid/Collection Stitching Plugin [44] implemented in ImageJ. We use the ‘unknown position’ option for the first time frame to calculate automatically the overlap between images, which we use for all frames because images are stable.

### 2.2. Data analysis

We measure the 2D velocity field ***v***(*x*,*y*,*t*) (figure 1*b*; electronic supplementary material, figure S1A,C) using particle image velocimetry [45]. We use the open source toolbox MATPIV *matpiv.m* [46] of Matlab (The MathWorks, Inc., Natick, MA, USA), with the ‘singlepass’ option, square box of side 32 pixels (20 μm) for 200 μm wide strips and 128 pixels (80 μm) for 1 mm wide strips, and box overlap is 50 or 75% for both widths.

The particle image velocimetry method, option ‘single’, interrogation box size of 128 pixels, yields qualitatively identical results, and is quantitatively around 10% larger, when compared either with ‘multin’ option, windowsize-vector [128×128;64×64] or with the Kanade–Lucas–Tomasi (KLT) feature-matching algorithm, pyramid parameter 2, successive interrogation box sizes of 128 and 64 pixels.

We do not detect any statistically significant dependence of ***v*** with *y*, even near the lateral sides of the strip. The *y* component of ***v***(*x*,*y*,*t*) is lower at higher positions *x* where the average velocity is higher (electronic supplementary material, figure S1C), indicating a more directed movement; we do not consider this component in what follows.

The component of ***v***(*x*,*y*,*t*) along the *x*-axis, i.e. along the long axis of the strip, averaged over *y*, is the 1D velocity field *V* (*x*,*t*), which we study here. This first step, projecting ***v*** on *x* and averaging it over *y*, is already enough to make visible the main features of the velocity field: velocity gradient along *x* and propagating waves.

To improve the visualization, and enable for a qualitative analysis, we plot the space–time diagram or ‘kymograph’ of *V* (*x*,*t*). The next step consists in filtering it. We first remove small-scale noise using a Gaussian blur of standard deviation 15 min and 30 μm (and a sliding window which is three times larger). We then separate scales, and decompose this de-noised *V* into large-scale $\bar{V}$ and middle-scale $V-\bar{V}$, using a Gaussian filter of standard deviation 50 min and 100 μm (again, with a sliding window which is three times larger).

For large-scale profiles $\bar{V}$, discrete measurements used for graphs are performed with an average over 176 μm wide space boxes in the distance *d* to the moving front (this coordinate *d* is oriented from the migrating front towards the cell reservoir, as opposed to the coordinate *x*); and an average on time *t* over 180 min. We entirely exclude the first box, where statistics are noisy due to the front. Only significant data points are plotted, i.e. points with enough pixels in the 176 μm×180 min space–time bin (at least 150 pixels, out of a maximum of 612) and where the signal value is larger than its s.d. value. For the velocity gradient, a finite difference gradient is used and the resulting very small-scale noise is removed with a 3-pixels-wide linear filter.

Similarly, by using histone-GFP (electronic supplementary material, figure S1B), we identify cell nuclei. Our measurements are based on local maxima detection, independently of the maximal intensity value, and thus are not sensitive to possible variations in the GFP signal intensity. The nuclei density can vary by a factor 10 within a given image (when including front cells). We first use a low-blur radius, optimized for the highest nucleus density on the image. It yields a good detection for high-density regions using *FastPeakFind.m* but gives false positives (more than one local maxima per nucleus) for low-density regions. When the distance between two maxima is smaller than a critical value (equal to a third of the local average distance between nuclei), we remove the less intense one. According to manual checks on high-, middle- and low-density regions, the precision is better than 5%.

We have checked that tracking the cell nuclei yields more fluctuations than PIV for ***v***(*x*,*y*,*t*) measurements, due to intracellular movements of nuclei (electronic supplementary material, movies S3–S6), but once projected and averaged, it yields same results as PIV for *V* (*x*,*t*). We use this cell nuclei detection to plot the cell density *ρ*(*x*,*t*), using boxes (*x*,*y*) of 20 μm×20 μm and then an average over *y*. Using the same filters as for *V* , we remove noise, decompose *ρ* into $\bar{\rho }$ and $\rho -\bar{\rho }$, and define discrete measurements of density with an average on time *t* over 180 min and on space over 176 μm wide boxes in the distance *d* to the moving front. We entirely exclude the first box, where statistics are noisy due to the front. In figure 5*c* and electronic supplementary material, figure S3B, the average near the front is taken on the three boxes (3×176=528 μm) next to the front one.

To perform quantitative analyses of the kymographs, we use wavelets as measurement tools (rather than as filtering tools). They extract from a signal its wavelengths and time frequencies, like Fourier transform does, but in addition wavelets can determine the space and time variations of these quantities. We use a custom-made software for wavelet transform profilometry (WTP), a method inspired by 3D-fringe projection profilometry [47,48]. It involves a 1D continuous wavelet transform with a phase estimation approach. It is reliable, easy to implement and robust to noise. We choose a Morlet wavelet and the wavelet transform is computed using an FFT algorithm (which is equivalent to an analytic Morlet Wavelet) with a Matlab script [49].

For each kymograph line (i.e. for each fixed position *x*_*i*_), the signal wavelet transform is computed at various time scales, in an observation window of 80–400 min, chosen in order to cover the full range of characteristic times of the observed oscillations (we checked that this choice does not affect the results presented here). The wavelet transform returns a matrix of complex coefficients *A*(*x*_*i*_,*t*,*s*), defined as continuous wavelet coefficients where *s* represents the test times scales. Each coefficient provides a local measurement of the similarity between the signal and the wavelet at a scale *s*. For each point (*x*_*i*_,*t*_*j*_) of a given line *i* in the kymograph, only the coefficient *A*_*m*_(*x*_*i*_,*t*_*j*_) having the largest modulus with respect to the scale *s* is kept.

The argument of *A*_*m*_(*x*_*i*_,*t*_*j*_) provides the wrapped fringe phase *ϕ*_*w*_(*x*_*i*_,*t*_*j*_). The phase *ϕ*_*w*_(*x*_*i*_,*t*_*j*_) is unwrapped along time, and the local angular frequency *ω* (2*π* times the frequency) is deduced by differentiation with respect to time *t* according to the sign convention *ω*=∂*ϕ*_*V*_/∂*t*. Independently, the phase *ϕ*_*w*_(*x*_*i*_,*t*_*j*_) is unwrapped along space, and the local wavenumber *k* is deduced by differentiation with respect to space *x* according to the sign convention *k*=−∂*ϕ*_*V*_/∂*x*.

## 3. Results

Section (a) reports and quantifies coherent collective cell migration over several millimetres. *A priori*, one could expect the cell velocity to depend on density, density gradient and distance to the front (hence on the monolayer history). In fact, the mean cell velocity profile in the monolayer bulk depends explicitly only on the cell density, irrespectively of the distance to the migrating front. It is very sensitive to proliferation and to lamellipodia inhibition. Section (b) reports that, on top of the average velocity and density profiles, backward propagating waves in density and velocity exist, and they have an opposite phase. We measure the local characteristics of the velocity waves and their variation in space. Their velocity decreases with cell density. Inhibiting lamellipodia formation damps the waves and decreases their frequency or even leads to their suppression.

### 3.1. Large-scale profiles of velocity and density

By averaging over the direction *y* perpendicular to the stripes, we determine the 1D cell velocity field *V* (*x*,*t*) and cell density field *ρ*(*x*,*t*). We first investigate their overall profiles $\bar{V}$, $\bar{\rho }$, obtained by large-scale sliding average. Owing to the spreading of cells, variations of velocity and density are visible (figure 1*b*). Far from the front (bottom of figure 1*b*), the density is still close to its initial value and the velocity is still zero, while close to the front (top of figure 1*b*) the density has decreased and the velocity increased. Note that far from the front, velocities are occasionally negative. We introduce *R*=(*πρ*)^−1/2^; then $R_{\mathrm{mean}}={\left(\pi \bar{\rho }\right)}^{-1/2}$, interpreted as a mean effective cell radius. Its typical range of variation is 8–15 μm; for comparison, note that 4.8 μm corresponds to the nuclei being almost close packed, while 17 μm is the radius of a front cell at the limit of detaching from the monolayer.

With respect to the distance *d* to the moving front, different experimental batches display $\bar{V}(d)$ profiles which are qualitatively similar and quantitatively different (figure 2*a*), and *R*_mean_(*d*) profiles too (figure 2*b*). When eliminating the space variable *d*, points coming from different batches fall on the same curve: $\bar{V}$ has a strong, negative correlation with $\bar{\rho }$ [13] (figure 2*c*; electronic supplementary material, figure S3A), decreasing from $0.8\;\text{μ}m\;\min^{-1}$ to 0 for $\bar{\rho }$ ranging from 1.8 to 5×10^−3^ μm^−2^. In fact, $\bar{V}$ increases linearly (with a non-zero intercept) with the mean effective cell radius, $\bar{V}=0.106\;R_{\mathrm{mean}}-0.864$ (figure 2*d*). This relation does not depend on the distance to the front, and is unaffected when the sliding window size in time is doubled.

We also performed experiments under standard conditions (i.e. without mitomycin C; electronic supplementary material, figure S4). While the overall relation between $\bar{\rho }$ and $\bar{V}$ seems qualitatively unaffected, dividing cells have a significantly larger $\bar{V}$ at given $\bar{\rho }$, and a larger ‘arrest density’ defined as the intercept of the velocity graph with the density axis: $\bar{V}$ decreases from $0.3\;\text{μ}m\;\min^{-1}$ to 0 for $\bar{\rho }$ ranging from 3 to 10×10^−3^ μm^−2^ (figure 2*c*; electronic supplementary material, figure S3A).

Inhibiting lamellipodia formation drastically decreases the monolayer average velocity (figures 2*c* and 4).

### 3.2. Propagating waves

**Figure 3.**
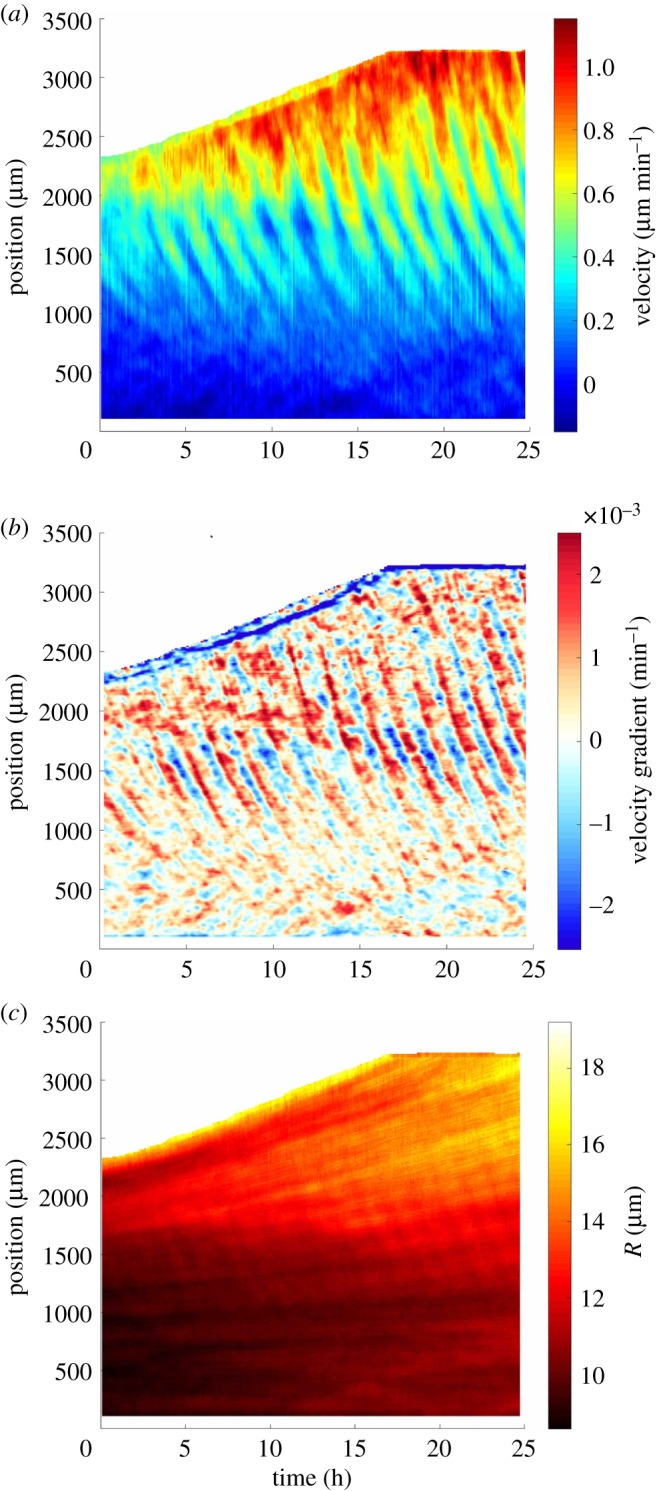
Propagating waves. Space–time diagram (kymograph) of (*a*) cell velocity *V* , (*b*) velocity gradient $\partial \tilde{V}/\partial x$ and (*c*) effective cell radius *R*. Space *x* is oriented from cell reservoir (bottom, 0 mm) to towards the front (top, 3 mm), time *t* from left (0 h) to right (25 h) and the top-left region is the bare substrate in front of the monolayer. All eight strips showed similar results.

We now turn to middle-scale variations. The cell velocity *V* (*x*,*t*) displays waves: cells slow down and accelerate while waves propagate from the front backwards in the −*x* direction (electronic supplementary material, movies S1–S6). In the moving frame of average cell velocity, these waves would appear as a periodic velocity reversal. They are visible quantitatively even on the raw kymograph (figure 3*a*; electronic supplementary material, figure S5A), and more clearly on the velocity middle-scale variations $\tilde{V}=V-\bar{V}$ (electronic supplementary material, figure S6A) as well as on the velocity gradient (figure 3*b*). The waves are reproducibly observed near the front, with a good signal-to-noise ratio over more than 10 periods for a whole observation duration of approximately 20 h. We do not detect any particular effect of the strip width on waves (cf. electronic supplementary material, figures S4B, S5A).

First measurements are manual. They indicate that the waves have a period around 2 h, their wavelength is around 1 mm. Their amplitude decreases with the distance to the front: waves are not apparent near the reservoir, where the cell density is as high as 5×10^−3^ μm^−2^ and the cell velocity vanishes. Where they are visible, their amplitude is steady in time, and large: it represents a relative variation in local velocity which typically ranges from 15 to 30% (electronic supplementary material, figure S5B), i.e. up to 60% crest to crest. Their velocity (indicated by the slope of the wave pattern) is of the order of 10 μm min^−1^, and with a sign opposed to that of the cell velocity (indicated by the slope of the front position). The wave pattern is visibly curved: this evidences that the wave velocity (phase velocity) is larger near the front than in the middle of the monolayer.

Density middle-scale variations are dominated by local heterogeneities, which are signatures of initial density fluctuations (cells do not significantly mix nor rearrange) over a typical length scale of 200 μm. More precisely, on the kymograph of *R* or $\tilde{\rho }=\rho -\bar{\rho }$ these fluctuations appear as bars which, near the front, are almost parallel to the front; far from the front, they are closer to horizontal (figure 3*c*; electronic supplementary material, figure S6B); in between, along the line drawn on electronic supplementary material, figure S6C which has a slope of $0.31\;\text{μ}m\;\min^{-1}$, we measure $\bar{V}=0.33\pm 0.02\;\text{μ}m\;\min^{-1}$ (s.d.). This proves that these fluctuations are advected at local velocity $\bar{V}$ along with the monolayer itself. In addition, and although they are less visible, it is clearly possible to distinguish waves on the density (figure 3*c*; electronic supplementary material, figures S5C and S6B), which have the same period as the velocity waves and are in phase opposition with them (electronic supplementary material, figure S6C,D). They have a small amplitude, with a relative variation ranging from 1 to 2% (electronic supplementary material, figure S5D), i.e. up to 4% crest to crest.

**Figure 4.**
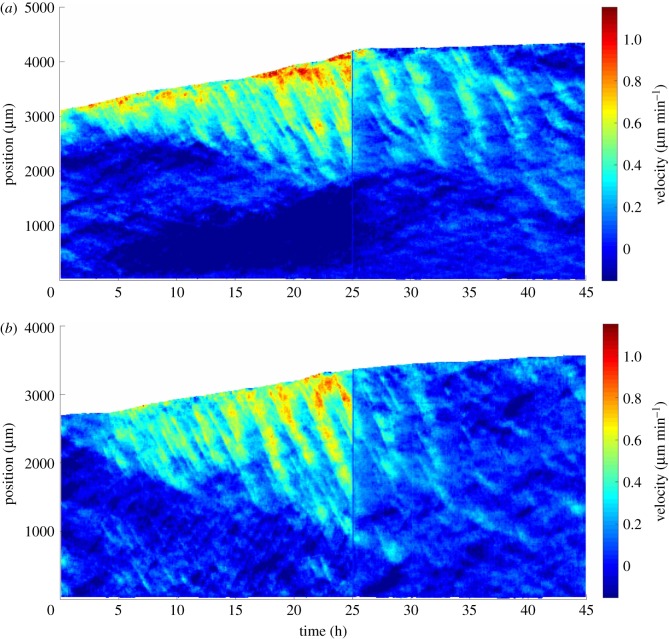
Propagating waves, same as figure 3, with application, after 1 day, of CK666 drug to inhibit lamellipodia formation, resulting in (*a*) attenuation of waves, *N*=7 strips; or (*b*) in their almost complete suppression, *N*=5 strips. Space *x* is oriented from the cell reservoir (bottom, 0 mm) to towards the front (top, 3 mm), time *t* from left (0 h) to right (45 h) and the top-left region is the bare substrate in front of the monolayer. The time of drug application (25 h) is visible as a vertical bar, because one image is not recorded.

Inhibiting lamellipodia formation strongly decreases the amplitude and frequency of velocity waves (figure 4*a*), sometimes even almost completely suppressing them (figure 4*b*). The wave velocity, visible as the slope of the wave patterns, is not significantly altered (figure 4*a*).

The wave pattern is visibly curved (figure 3*a*; electronic supplementary material, figure S6A): wave characteristics vary with space, and this can be quantified in several regions where the signal-to-noise ratio is sufficient (electronic supplementary material, figure S5A). Using wavelets, we define, distinguish and measure locally $\left|\tilde{V}\right|$ and *ϕ*_*V*_ at each position *x* and time *t*, as follows. The smaller space and time scales variations of $\tilde{V}$ are encompassed by the wave phase *ϕ*_*V*_ (electronic supplementary material, figure S7B), the larger space and time scales variations are encompassed by the wave amplitude $\left|\tilde{V}\right|$, with V~=Re(|V~|expiϕV). The wave amplitude $\left|\tilde{V}\right|$ tends to increase with the cell velocity $\bar{V}$ (electronic supplementary material, figure S7A) and accordingly decrease with the cell density $\bar{\rho }$.

The local phase *ϕ*_*V*_, in turn, determines by differentiation the local angular frequency *ω* and wavenumber *k*. The wave velocity *c*=*ω*/*k* is negative here because wave and cell velocities are in opposite directions, while we have chosen the convention that *V* >0. Hence *ω* and *k* are of opposite signs, and with our convention *ω*>0 while *k*<0. The local time period is *T*=2*π*/*ω* and the local wavelength is *λ*=2*π*/|*k*|.

We observe (figure 3*a*; electronic supplementary material, figure S6A) that *k* varies in space; conversely, at a given time, *ω* does not vary significantly with space. Accordingly, *ω* does not depend explicitly on local density, which varies significantly with space. Interestingly, comparing experiments with different density profiles shows that, in a 180 min× 528 μm bin near the moving front, the wave frequency decreases with the cell density (electronic supplementary material, figure S3B). As already observed qualitatively (figure 4*a*), *c* is of the order of minus 10 times the cell velocity $\bar{V}$ (cf. electronic supplementary material, figures S3A and C) and decreases with the mean effective cell radius *R*_mean_ (electronic supplementary material, figure S3C), i.e. |*c*| decreases with $\bar{\rho }$.

Again using wavelet analysis, we define and measure $\left|\tilde{\rho }\right|$ and *ϕ*_*ρ*_: ρ~=Re(|ρ~|expiϕρ). The kymograph of *ϕ*_*ρ*_ shows that the wavelets detect the signal which physically corresponds to the wave velocity (electronic supplementary material, figure S7B).

As already observed qualitatively (figure 4*a*), inhibiting lamellipodia formation significantly decreases *ω* (electronic supplementary material, figure S3B).

## 4. Phenomenological description

Continuum mechanics [50] has been successfully used to model collective migration and wound closure of a cell monolayer on a substrate [51,52]. Several models have been proposed to explain the instability which gives rise to waves by invoking one of various active cell ingredients, within the constraints raised by symmetry considerations [11,32,53–57].

Building on these models, we propose a simple phenomenological description (which means we model the phenomena without explicitly modelling their microscopic or biochemical causes). An advancing front cell pulls on its follower, which becomes polarized after some time delay, enabling signal propagation from the front backwards into the bulk; when the follower eventually increases its velocity, the front cell is free to increase its velocity too, generating time oscillations.

Our goal is to perform testable predictions, compare them with experiments and extract the values of relevant physical parameters. Continuum mechanics helps here to understand the physical effect of each parameter and draw a phase diagram. Numerical simulations, which could turn useful, for instance, to vary boundary conditions or to link cell-level ingredients with collective migration, are beyond the scope of the present work.

We first recall cell number and momentum conservation laws within continuum mechanics, here expressed in one dimension (§(a)), and couple them with the monolayer mechanical properties (§(b)). We then include an active force linked to cell polarization (§(c)) and a phenomenological coupling between strain and polarity (§(d)), to explain the existence of waves (§(e)) and predict their characteristics (§(f)).

### 4.1. Conservation laws

In the absence of cell division, the cell number conservation is expressed as

4.1 $$\frac{\partial \rho }{\partial t}+\frac{\partial (\rho V)}{\partial x}=0.$$

For simplicity, we develop a local model, neglecting the large-scale gradients. We introduce the angular frequency *ω*, the wavenumber *k*<0 and the wave velocity *c*=*ω*/*k*<0, and we treat them as numbers rather than as fields. As explained above, we separate the velocity into its average $\bar{V}$ and its variations $\tilde{V}$, the density into its average $\bar{\rho }$ and its variations $\tilde{\rho }$, and again we treat $\bar{V}$ and $\bar{\rho }$ as numbers rather than as fields. Within this approximation, wavelet analysis and Fourier analysis become indistinguishable.

We linearize equation (4.1) for small wave amplitude, i.e. neglecting the $\tilde{\rho }\tilde{V}$ term. It is written as $\omega \tilde{\rho }-k(\bar{V}\tilde{\rho }+\bar{\rho }\tilde{V})=0$, or equivalently after division by *k*:

4.2 $$\tilde{\rho }(c-\bar{V})=\bar{\rho }\tilde{V}.$$

As the order of magnitude of *c* is $-10\;\bar{V}$ (electronic supplementary material, figure S3A,C), equation (4.2) predicts that $\tilde{\rho }$ is in phase opposition with $\tilde{V}$ and that $\tilde{\rho }/\bar{\rho }$ is of the order of $-0.1\;\tilde{V}/\bar{V}$. This explains why density oscillations are barely visible (figure 3*c*; electronic supplementary material, figures S5C, S6B). By measuring the velocity and density wave characteristics at several points, we observe a local variability, which we exploit to check over a wide range that equation (4.2) is compatible with the observed oscillation amplitudes (electronic supplementary material, figure S8A): equation (4.2) is checked with 10% precision. Phases of $\tilde{\rho }$ and $\tilde{V}$ should differ by *π*, according to equation (4.2), which is checked up to better than 0.03 rad, or 1% precision (electronic supplementary material, figures S6D, S7B, S8B).

We now turn to the momentum conservation law, namely the force balance. The force equilibrium of the monolayer (integrated along the normal to the substrate) relates the external force per unit area *F*, exerted by the substrate on the cell monolayer, and the internal forces, namely the divergence of stress, as

4.3 $$\frac{\partial (h\sigma )}{\partial x}+F=0.$$

Here *σ* is the 1D stress (equivalent to the 3D stress component along *xx*) averaged over the monolayer thickness *h*. For simplicity, displacement and stress fields are assumed to be functions of *x* and *t* only and we consider only one component of each field in state equations, the component along the *x* direction. In a real 2D description of stress, this would have to be replaced by the deviator of the stress tensor; alternative possibilities exist, such as the mean of the two principal stresses within the cell monolayer, i.e. half the trace of the stress tensor [32].

### 4.2. Monolayer mechanical properties

In principle, the dissipation could be of both intra- or intercellular origin, and contribute to stress both in series or in parallel with elasticity [50]. These different monolayer rheological properties are compatible with the appearance of waves [57], and it is beyond the scope of the present paper to enter into such detailed description. To fix the ideas, the monolayer is often described as a viscoelastic liquid [11,51], with a dissipative contribution in series with the elasticity (Maxwell model) and an elastic strain which relaxes over a viscoelastic time *τ*:

4.4 $$\frac{de}{dt}+\frac{e}{\tau }=\frac{\partial V}{\partial x}$$

and

4.5 σ=Ge.

Here, in such a 1D Maxwell model, the velocity gradient ∂*V*/∂*x* (plotted in figure 3*b*) is equivalent to the total strain rate, which in turn is the sum of the elastic and viscous strain rates. They are in series, and the viscous strain rate is *e*/*τ*, where *τ* is the viscoelastic time and *e* is the elastic strain. It would be beyond the scope of the present paper to relate subcellular ingredients with this elastic strain *e*, which we consider here as an effective, coarse-grained variable [58]. The elastic strain rate is *de*/*dt*=∂*e*/∂*t*+*V* ∂*e*/∂*x*; *G* is the elastic modulus, typically in the range 10^2^–10^3^ *Pa*, obtained for a single cell [59], by micro-indentation [60,61] or on a monolayer [62] (note that stretching a suspended monolayer, including cell–cell junctions, yields a much larger value, approximately 2×10^4^ Pa [12]); the value of *τ* is discussed below. From these orders of magnitude, we predict that detecting waves of traction force should be technically challenging.

### 4.3. Active mechanical ingredients

In the literature, the force per unit area exerted by the substrate on the monolayer is often expressed as the sum of active and friction contributions, for instance *F*=*f*_a_*p*−*ζV* [14]. Here *f*_a_ is the characteristic value of the active force a cell can exert; it is of the order of 300 Pa [5] and decreases with *ρ* [7,8,13]. The dimensionless real number *p* is a mathematical term reflecting, within the current simplified 1D description, the actual 2D cell polarization. It is convenient to introduce *V*_a_=*f*_a_/*ζ*, which corresponds to a characteristic scale of active migration velocity, and write

4.6 $$\frac{F}{\zeta }=V_{a}p-V.$$

Note that, alternatively, it would have been possible to consider *p* as a Boolean variable, being either +1 or −1, while *V*_a_ and *f*_a_ would be continuous variables. This alternative could be important when discussing, for instance, how the polarity *p* is related with biochemistry, and whether it could change sign by passing continuously through 0; but such debate is beyond the scope of the present paper.

To fix the ideas, we use the values of the friction coefficient *ζ*∼10^9^ N m^−3^ s [14,32]. For a wavenumber *k*∼10^4^ rad m^−1^, and with upper estimates of the 3D cell viscosity *η* of order of 10^2^ Pa s [63,64], we obtain that the modulus of the internal viscous force $\eta k|\tilde{V}|$ is at least 1000 times smaller than that of the typical external friction force, $\zeta \bar{V}$. We thus neglect the viscosity contribution in parallel with the elasticity [32].

Combining equations (4.3), (4.5) and (4.6) to eliminate *F* and *σ* yields ∂_*x*_(*Ghe*)+*V*_a_*p*=*V* . Differentiating it and eliminating ∂_*x*_*V* with equation (4.4) yields a second-order differential equation in *e*:

4.7 $$\frac{de}{dt}=\frac{\partial e}{\partial t}+V\frac{\partial e}{\partial x}=\frac{\partial^{2}(De)}{\partial x^{2}}+\frac{\partial (V_{a}p)}{\partial x}-\frac{e}{\tau }$$

A typical range of variation of *h* is from 13 μm far from the front to 8 μm near the front (see Supp. Fig. S7 of Serra-Picamal *et al.* [11], where the cell volume is approximately conserved). As *h* varies slowly with space, equation (4.7) is locally a diffusion-like equation [65], where the effective strain diffusion coefficient is *D*=*Gh*/*ζ*; *D* increases with monolayer stiffness and decreases with friction. In principle, the steady flow can become unstable, and waves appear, if the heterogeneity of the active term *V*_a_*p* overcomes the stabilizing diffusion term *D*. A heterogeneity in migration force might create a heterogeneity in velocity, affecting in turn the stress, which would feed back on the force. The question is how this feedback could become positive and strong enough to make the flow unstable.

### 4.4. Strain–polarity coupling equation

Polarity can couple to cell strain through a mechano-sensitive protein or protein complex (such as Merlin [66]). Here we assume that the monolayer is already polarized, symmetry is broken due to the migrating front (hence symmetry constraints [56] are not enforced in the following equations). Cells already have a polarity *p*, which is enhanced by cell stretching and decreased by cell compression. We neglect: nonlinearities; viscosity; and the large-scale variation of *ρ* and $\bar{V}$ over the whole strip length scale. These simplifying hypotheses can easily be relaxed if required, for instance, if future experiments add new details. We have checked a posteriori that a complete treatment which includes the space variations of *ρ* and $\bar{V}$ modifies the present predictions of |*c*| and *ω* by less than 10%.

We study the stability of a homogeneous steady state where all cells migrate in the same direction, $\bar{V}=V_{a}>0$, and are positively polarized, $\bar{p}=1$. The density is $\bar{\rho }$, the traction force $\bar{F}$. We study, for instance, the case $\bar{e}=0$, which is relevant in the region close to the front where the waves are most visible, and which is the value towards which *e* relaxes when *τ* is finite. Note that the mirror-reflected steady state, where $\bar{V}$ and $\bar{p}$ would be negative, is irrelevant here (unlike in symmetric migration experiments [11]). The third steady state, $\bar{V}=0$, exists initially, but ceases to be stable when the confinement is removed, and is also irrelevant here.

The polarity follows the strain with a delay *τ*_p_ reflecting, within the current simplified 1D description, the actual 2D amplitude and orientation relaxation time [51]:

4.8 $$\frac{\partial p}{\partial t}=\frac{1+me-p}{\tau_{p}}.$$

Here *m* is a non-dimensional factor coupling polarity and cell strain, and when *me* is of order 1 the polarity value changes by one unit. If we integrate equation (4.4) in time, using the observed velocity wave characteristics, we find that the total strain has an amplitude of order 0.1. It means that the elastic strain has an amplitude of at most 0.1, and probably around half of it if *de*/*dt* and *e*/*τ* are comparable (which is the case, because *τ* is comparable with the wave period, see below). Hence a strain of at most 0.1 (and possibly 0.05) suffices to change the polarity from value 0 to value 1, and *m* is of order of 10, possibly 20, or 25 at most.

### 4.5. Onset of wave appearance

To perform a linear stability analysis around the steady state $\bar{e}=0$, $\bar{p}=1$, the small variables are *δe*=*e* and *δp*=*p*−1. Terms due to variations of *h* are of second order and thus negligible. Equations (4.7) and (4.8) become, after linearization:

4.9 $$\begin{array}{rl} \frac{d\delta e}{dt} & =D\frac{\partial^{2}\delta e}{\partial x^{2}}+V_{a}\frac{\partial \delta p}{\partial x}-\frac{\delta e}{\tau }\\ \;\text{and}\;\tau_{p}\frac{d\delta p}{dt} & =m\delta e-\delta p. \end{array}\}$$

We look for small perturbations (of the steady, homogeneous state) proportional to exp⁡(st−ikx), where the wavenumber *k* is a real number, and the wave growth rate *s* is a complex number with a real part which is strictly positive when the steady state is unstable, Re(*s*)>0, and a non-zero imaginary part, Im(*s*)≠0. The Jacobian matrix of the equation system (4.9), is

4.10 $$\left(\begin{array}{cc} s-ik\bar{V}+Dk^{2}+\tau^{-1} & ikV_{a}\\ -m & \tau_{p}(s-ik\bar{V})+1 \end{array}\right).$$

**Figure 5.**
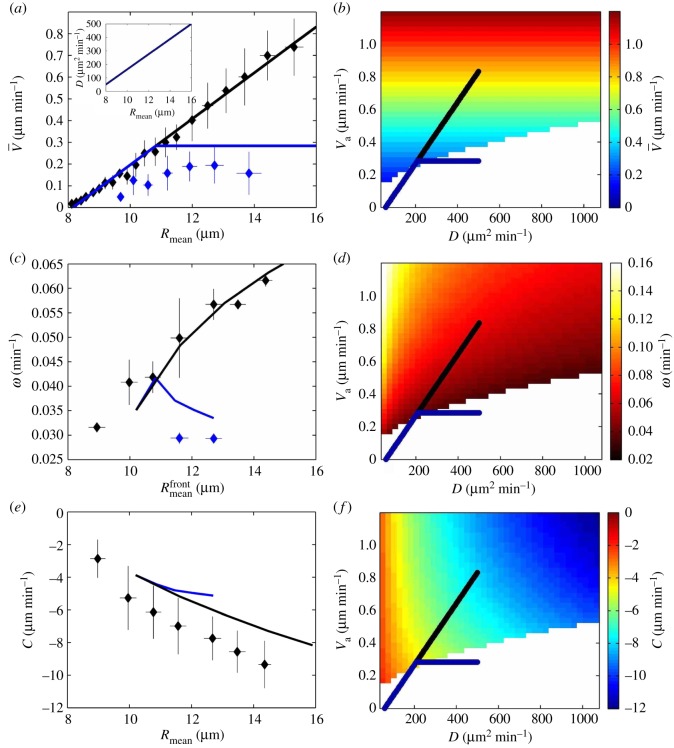
Predictions of the phenomenological model. Realistic parameter values (strain–polarity coupling term *m*=25, polarization delay time $\tau_{p}=15\;\min$, viscoelastic time τ=180 min) are manually chosen to obtain a good agreement with the data (see text). (*a*) Large-scale average of cell velocity $\bar{V}$ versus mean effective cell radius *R*_mean_. Points are experimental data from figure 2*c*,*d*. Black: experiments with mitomycin; black line: linear relation; blue: experiments with lamellipodia inhibition using CK666 drug; blue line: we draw a linear increase followed by a plateau (see text for details). Inset: *D* versus *R*_mean_, estimated relation, not affected by CK666. (*b*) Diagram of cell velocity $\bar{V}$ versus strain diffusion coefficient *D* and active velocity *V*_a_. It is plotted in the region where the wave amplitude growth rate is positive (existence of waves). The regions where the wave amplitude growth rate is negative (the steady migration is stable) are left blank. Lines correspond to those in (*a*); *R*_mean_ is increasing from bottom left to top right. (*c*,*d*) Same for wave angular frequency *ω*, which depends on the mean effective cell radius; they are measured in a 180 min×528 μm bin near the moving front (electronic supplementary material, figure S3B). (*e*,*f*) Same for wave velocity *c*; note that its values are negative, and that with CK666 drug the values of *c* are too noisy for quantitative measurements, but are similar.

Solving in *s* simply requires one to write that the determinant of J is zero:

4.11 $$\tau_{p}S^{2}+\left(1+\tau_{p}Dk^{2}+\frac{\tau_{p}}{\tau }\right)S+Dk^{2}+\frac{1}{\tau }+imkV_{a}=0,$$

where $S=s-ik\bar{V}$. There are two roots, which depend on *k* and on parameter values. We numerically solve equation (4.11) and look for a root with Re(*s*)>0 and Im(*s*)≠0. Depending on the parameter values (figure 5), there exists a range of *k* with one such root, and the steady, homogeneous solution is unstable. A propagating wave appears; the mode *k* which develops more quickly is the one for which Re(*s*) is maximum, i.e. d Re(*s*)/d*k*=0 (until the amplitude increases enough to reach the nonlinear regime). Its imaginary part Im(*s*) is the angular frequency *ω* of this mode.

To fix the ideas and provide example of calculations, we take from experiments that $\bar{V}$ is of the order of $1\;\text{μ}m\min^{-1}$. The delay time *τ*_p_ of polarity with respect to stretching, due to the reaction time of the Rac pathway, could be of the order of 25 min [66]. The value of *m*, ranging from 10 to 25, also affects the predictions; higher *m* values tend to make the absolute value of *c* larger (*c* is more negative). We defer the discussion of *V*_a_ and *D* to §(f) and now discuss the value of *τ*.

In the viscoelastic liquid description we consider here, at time scales smaller than *τ* the monolayer can sustain a shear stress and behaves as mostly solid, while at longer time scales the strain relaxes and the monolayer behaves as mostly liquid. So it is important to determine whether *τ* is larger or smaller than the time scale of the waves, which is of the order of 1 h. This is subject to debate, because, depending on the cell line, the elastic modulus and the viscoelastic time of tissues can vary over orders of magnitude [58]. Even when restricting to MDCK monolayers, published values for the viscoelastic time *τ* range from 15 min [51] to 3–5 h [58]. Several articles [5,11,52] choose to treat the monolayer as elastic, given that the elastic modulus can be an effective modulus arising from the cell activity [58,67].

We have checked numerically that small values of *τ*, for instance 30 min or less, stabilize the steady state, while large values of *τ*, 1 h or more, allow for the wave appearance. The wave characteristics we determine barely change when *τ* spans the range 1–5 h.

### 4.6. Wave characteristics

The active cell velocity *V*_a_ makes waves appear, while the strain diffusion coefficient *D* damps them. Experimental measurements of wave characteristics can help estimate orders of magnitude of *D* and *V*_a_. However, determining their precise values is difficult and strongly dependent on the model (which is here only phenomenological). We thus let values of *D* and *V*_a_ vary within a reasonable range and solve systematically equation (4.11), to determine a phase diagram in the (*D*,*V*_a_) plane.

In figure 5, we plot the model predictions for manually chosen, realistic parameter values $\tau_{p}=15\;\min$, τ=180 min and *m*=25. The experimental data indicate that both *V*_a_ and |*c*| increase linearly with *R*_mean_ (figures 2*d* and 5*e*; electronic supplementary material, figure S3C). We use the measured relation $\bar{V}=0.106\;R_{\mathrm{mean}}-0.864$ (figures 2*d* and 5*a*). To reproduce the experimentally observed linear variation of |*c*| with *R*_mean_ (figure 5*e*), we find that *D* too has to increase with *R*_mean_; in the following we choose a linear relation between *D* and *R*_mean_ (inset of figure 5*a*). This determines the black lines on figure 5*b*–*f* as possible variations of $\bar{V}$, *c* and *ω*, and corresponding trajectories in the (*D*,*V*_a_) plane, when *R*_mean_ varies. We obtain an agreement with measurements of *ω* versus *R*_mean_ at the front, which we reproduce quantitatively (figure 5*c*), and of *c* versus *R*_mean_, which we capture qualitatively (figure 5*e*). Note that *c* is negative, indicating backward waves as observed in experiments.

The instability threshold, visible as the limit between coloured and blank regions (figure 5*b*,*d*,*f*), indicates when *V*_a_ is strong enough to overcome *D*. We obtain a consistent picture with typically *D* of the order of $10^{2}\;\text{μ}m^{2}\;\min^{-1}$ (i.e. *G* of a few 10^2^ Pa), *V*_a_ of the order of $10^{-1}\;\text{μ}m\;\min^{-1}$ (*f*_a_ of the order of one or a few 10 Pa), wavelength of several 10^2^ or 10^3^ μm, time period of a few $10^{2}\;\min$ and |*c*| of the order of $10^{1}\;\text{μ}m\;\min^{-1}$.

We observe that CK666 drug treatment results in a saturation in $\bar{V}$, probably linked with a limit in lamellipodia size [43] (figure 2*c*). To position the corresponding predictions (blue lines in figure 5) without introducing free parameters, we proceed as follows. As within the model $V_{a}=\bar{V}$, we use at small *R*_mean_ values the same linear increase as without CK666 (figures 2*d* and 5*a*), and at larger *R*_mean_ values the saturation corresponds to a plateau. To determine the position of the crossover between these regimes, i.e. the onset of saturation, we observe that in experiments with the CK666 drug, the monolayer is very close to the limit of wave appearance: it can lead either to decrease in wave amplitude and frequency, or to a suppression of the wave. We thus position the crossover at the intersection between the straight line and the instability threshold; this qualitatively captures all features of figure 5 without any additional free parameter.

Experimentally, in the case where small waves are observed in presence of CK666 drug, then *R*_mean_ is near 12 μm and *ω* is near $0.03\;\min^{-1}$. In this case, the wave velocity *c* measurement is too noisy to be quantitative, but *c* seems unaffected as no slope rupture is visible on the wave pattern (figure 4*a*). These features are qualitatively captured by our model.

## 5. Discussion

We first discuss the average velocity profile and the fact that it depends on density rather than on the distance to the moving front; our quantification gives evidence of the effect of proliferation and the key role played by polarity in cell migration (§(a)), as well as in velocity waves (§(b)). Our experiments and our phenomenological model agree reasonably, thus improving our understanding (§(c)), and contributing to explain recent observations of backward waves in colliding monolayers [29]. More broadly, our experimental data help to discriminate between existing theories (§(d)).

### 5.1. Cell migration results

Experiments had shown that *V* decreased with the distance *d* to the moving front [11], while *ρ* and *σ*_*xx*_ increased with *d* and were proportional to each other [5]. We can imagine two possible interpretations: either that both *ρ* and *σ*_*xx*_ happen to vary similarly with *d*, with a reinforcement of cell–cell junctions from the front to the back; or that *σ*_*xx*_ is actually determined by *ρ*.

Here, we observe a large enough range of cell densities *ρ* and velocities *V* , and of distances *d* to the front, with a good enough signal-to-noise ratio, to discriminate between *V* depending on *d* versus on *ρ*. We find that, in the monolayer bulk, *V* depends only on *ρ*, namely that it increases linearly with *R*_mean_, irrespectively of *d* or of the past history of the cell monolayer (advection, divisions, extrusions) which causes the observed density value. This is compatible with observations of Trepat *et al.* [5] and Serra-Picamal *et al.* [11]; it suggests that the traction force is cell-autonomous and is linear in *R*_mean_; and it favours the interpretation that *σ*_*xx*_ is determined by *ρ*.

If the monolayer spreading was only determined by stretching under a stress gradient, then it would be similar to a passive material wetting a solid substrate; the velocity and density profiles would depend on the distance to the front. Our experiments rule out this description: indeed, the velocity profile rather depends directly on the density, and the cell-autonomous active term of equation (4.7), ∂(*V*_a_*p*)/∂*x*, plays an important role. Conversely, the stretching under a stress gradient actually feeds back on the cell polarity and activity, through equation (4.8) and its non-autonomous signalling term *me*. We suggest that this mutual coupling between cell-autonomous active migration and non-autonomous stretching by neighbouring cells, summarized by equation (4.9), gives rise to collective migration.

Oriented cell divisions have two antagonistic effects (figure 2*c*; electronic supplementary material, figure S4). First, they contribute to increase the cell movement and the front velocity; they also increase the noise in cell velocity, and more regions have a negative velocity (electronic supplementary material, figure S4D). Hence at a given density, a proliferating monolayer has a higher velocity than a non-proliferating one. Second, however, a monolayer with a high proliferation rate has a different time evolution: its density increases with time (while a monolayer with a low proliferation rate or no proliferation at all has a density which decreases with time due to spreading). A monolayer with a high proliferation rate has a velocity which decreases with time and reaches within hours a density where cells are jammed and lamellipodia are absent, the front velocity is low and mainly due to divisions.

With migration alone, in the absence of division, coherent cell collective movement propagates over several millimetres inside the monolayer. The wavelength is of order of 1 mm and more broadly speaking the whole velocity field is established coherently over 4 mm. We have used 200 μm and 1 mm wide strips, larger than the typical 200 μm correlation length for the cell velocity field [9,28]. We do not detect any significant effect of the strip width on the results presented here.

Our observations are compatible with a large-scale polarized activity induced, for instance, by activity of the protein Merlin (a tumour suppressor), as recently shown experimentally [66]. The cells at the front of the migrating monolayers are known to exert large traction forces [62,68] that can induce the build-up of a large intercellular stress and, in turn, a polarization of the following cell by a relocalization of Merlin from the cell–cell junctions to the cytoplasm. When the cell is at rest, Merlin is localized at the cell–cell junctions. This junctional Merlin inhibits the formation of cryptic lamellipodia. On the other hand, when cell–cell junctions experience a stretching stress, Merlin is relocated to the cytoplasm. Owing to the decrease in junctional Merlin, the Rac pathway becomes activated, within a delay time of a few tens of minutes. Then, within a much smaller delay, Rac activates the generation of cell polarization and lamellipodia, responsible for the migrating forces [66]. The iteration of such processes may lead to large-scale polarization within the tissue.

To complement existing experiments with blebbistatin which focus on the role of cell contractility [11,32], we inhibit lamellipodia with CK666 drug treatment. It has a clear effect, even in the bulk of the monolayer, on *V* , which is decreased; and on the *V* (*R*_mean_) relation, which saturates and is no longer linear (figure 2*c*). This suggests that the contribution of lamellipodia to the traction force is dominant, and linear in *R*_mean_.

### 5.2. Wave results

Our observations and our model agree with experimental observations by Trepat and co-workers (Fig. 3a,b of [11] and [29]) that waves arise at the front and propagate backwards, with the wave velocity direction opposed to the cell velocity direction.

In experiments with divisions, a monolayer with a high proliferation rate leaves too quickly, or never reaches, the low-density regime where large-amplitude steady waves develop. The mechanical waves are slightly visible and overdamped; this is broadly compatible with the literature [11,30–32].

Without divisions, we obtain a good enough signal-to-noise ratio to measure the wave properties. Moreover, owing to this signal-to-noise ratio and experiment reproducibility, we can even measure the variation of wave properties across space and time, and with enough precision to discriminate between dependence with position versus with density. We observe that the wave velocity *c* is of the order of $-10\;\bar{V}$ and, like $\bar{V}$, it depends explicitly only on *ρ*: it is linear in *R*_mean_, again irrespectively of distance *d* to the moving front or of the past history of the cell monolayer.

For a given experiment, although *ρ* is space-dependent, the wave angular frequency *ω* is spatially homogeneous (figure 3*a*; electronic supplementary material, figure S6A). This might result from the most developed mode temporally forcing the instability over the whole monolayer. As a consequence, the wavenumber *k* depends on space. Now, comparing experiments at different densities, we observe that *ω* increases with *R*_mean_ measured near the front (figure 5*c*; electronic supplementary material, figure S3B). Inhibiting lamellipodia formation decreases *ω*, at a given value of *R*_mean_ measured near the front (electronic supplementary material, figure S3B).

Backward propagating waves are reminiscent of a generic instability mechanism originally discussed in the context of car traffic [69], which arises when the velocity *V* is a decreasing function of density *ρ*. For instance, velocity pulses have been observed for dense colloids in channel flow near jamming–unjamming transitions, in experiments [70] and in simulations [71]. Similarly, self-propelled agents, which tend to accumulate where they move more slowly and/or slow down at high density (for either biochemical or steric reasons), undergo a positive feedback which can lead to motility-induced phase separation between dense and dilute fluid phases [72,73].

### 5.3. Comparison of experiments with phenomenology

Inspired by published observations and by our own, we propose here a simple description where motility forces in the bulk of a homogeneous monolayer are oriented by a dynamic pulling on cell–cell junctions. This elasticity–polarity coupling is combined with classical rheology equations of continuum mechanics.

Our experimental observations with drugs against proliferation or lamellipodia are compatible with the theoretical picture in which waves spontaneously appear close to the instability limit (figure 5). This could explain why in preceding experiments with proliferation and jamming, waves were damped and more difficult to extract from noise beyond one period [11,30,31] (see also the Supp. Figs. S2, S5 of Notbohm *et al.* [32]). It also explains that wave observations are sensitive to experimental details; parameters can change from one experiment to another depending on cell size or substrate properties such as stiffness or coating.

With reasonable parameters values, typically *G* of a few 10^2^ Pa, *f*_a_ of one or a few 10 Pa, we find propagating waves with wavelength of several 10^2^ or 10^3^ μm, time period of a few $10^{2}\;\min$, of the same order as the experimental values. We predict a negative wave velocity *c* indicating backward waves, with |*c*| of the order of $10^{1}\;\text{μ}m\;\min^{-1}$, as observed in experiments. This backward propagation can probably be explained because a cell migrating towards the front pulls the cell behind it and favours its migration (in our model, under traction the cell polarity increases, with a positive coupling factor *m*).

We expect that when *ρ* decreases (*R*_mean_ increases), *V*_a_ significantly increases (figure 5). It is compatible with our observation that, when comparing experiments at different densities, *ω* decreases with *ρ* (figure 5*c*). Our model presents a Hopf bifurcation sensitive to the density, and a slowing down of the wave frequency when approaching the bifurcation. With proliferation due to cell division, the density increases; it can lead either to decrease in wave amplitude and frequency, or to a suppression of the wave, as observed in experiments (figures 4 and 5*c*). The model suggests that two experiments performed at a slightly different initial density can, after lamellipodia inhibition, lead either to decrease in wave amplitude and frequency, or to a suppression of the waves; this could explain the observed effects of CK666 drug (figures 4 and 5*c*).

Our model qualitatively suggests that *D* increases with *R*_mean_ (figure 5*a*, inset). Our estimation of *D*, which increases linearly by a factor of 10 when *R*_mean_ doubles (figure 5*a*, inset), is compatible with the observation that the elastic modulus can vary over orders of magnitude, and scales linearly with the size of the constituent cells [58]. This could be compatible with the intuition that a cell which spreads has more stress fibres and a more organized cortex, resulting in a larger cell stiffness *G*. It is also compatible with the fact that the relation *D*(*R*_mean_) is much less influenced than the relation *V*_a_(*R*_mean_) by the lamellipodia inhibition. Note that alternative explanations of *D* variations with cell size exist, for instance if the friction coefficient *ζ* was decreasing with *R*_mean_.

In summary, our model (figure 5) is precisely compatible with measurements of $\bar{V}$, reproduces quantitatively *ω* and qualitatively *c*, whose sign is correctly predicted. Our predictions of how *V*_a_ and *D* vary with *R*_mean_ agree with independent experiments on the same MDCK cells [58]. In the presence of CK666 drug, our model is qualitatively compatible with either the suppression of waves, or waves with a smaller amplitude and frequency and an unchanged velocity.

### 5.4. Comparison with existing models

Marcq and co-workers have shown that as cells are active, the appearance of waves is compatible with a wide range of ingredients. In particular, the rheology is not crucial, as waves can appear in materials with various rheological behaviours [54,57]. They predict that there are more waves when divisions are inhibited [56] and less waves when lamellipodia are inhibited [57]. Our observations and model agree with these predictions.

Banerjee, Marchetti and co-workers [32,53] predict the existence of waves depending on an effective elasticity and traction force amplitude. Our model is based on an approach similar to theirs; we make it simpler while keeping the main ingredients. They find a wave velocity comparable to a cell length divided by the time (approx. 2 min) required for mechanical stress information to propagate across the cell. Their wave period, around 6±2 h, increases with decreasing traction forces. Their predictions are consistent with three of our observations. First, their waves propagate backwards. Second, the wave frequency increases with increasing density (and thus decreasing traction force) at the migrating front. Third, when adding CK666 drug, either the wave frequency decreases or the wave disappears.

Blanch-Mercader & Casademunt [55] explain that even viscous tissues can have an effective elasticity in which waves appear. This is compatible with the idea, arising from both experiments [58] and models [67], that the elastic modulus can be an effective modulus arising from the cell activity.

Serra-Picamal *et al*. present numerical simulations [11] where, based on their cell-stretching experiments, they introduce a dynamically changing elasticity (nonlinear cytoskeletal reinforcement). Our ingredients are similar to theirs except that, inspired by recent experiments on collective cell migration [66], we introduce a dynamically changing traction force modulated by the elastic strain.

Note that, in Fig. 4d–f of Serra-Picamal *et al.* [11], simulations predict forward-propagating waves, arising at the centre and moving towards the front, i.e. waves velocity in the same direction as cell velocity; Fig. 6 of Blanch-Mercader & Casademunt [55] also predicts forward-propagating waves. By contrast, experiments in [11,29], our experiments and our model agree that waves arise at the front and propagate backwards. We are not aware of any interpretation of this discrepancy.

## 6. Conclusion

In summary, by inhibiting cell proliferation in a cultured epithelial cell monolayer, we limit density increase and observe steady migration over a day or more, without reaching jamming densities. We observe for the first time a coherent collective cell migration propagate over several millimetres; cells spread and the density decreases from the monolayer bulk towards the front. Cell velocity increases linearly with the cell effective radius, and does not depend directly on the distance to the front.

On top of this average velocity profile, we detect 10 periods of backward-propagating velocity waves, with millimetric wavelength. The signal-to-noise ratio is sufficient to perform precise and reproducible measurements of local characteristics of the wave and their spatial variation. Their velocity (approx. 10 μm min^−1^) is 10 times the cell velocity; it increases linearly with cell radius. Their period (approx. 2 h) increases with the cell density at the front. The period is spatially homogeneous, which might result from the most developed mode temporally forcing the instability over the whole monolayer. As a consequence, the wavenumber depends on space. Density waves also appear, with a tiny amplitude and a phase opposed to that of velocity waves.

The most visible effect of cell divisions is to steadily increase cell density, which contributes to jamming and decreases the migration velocity. However, at a given density, divisions contribute to increase front velocity, cell velocity and noise in cell velocity. When we inhibit lamellipodia formation, cell velocity drops while waves either disappear, or have a smaller amplitude and slower period. Our results suggest that the lamellipodia contribution to the cell traction force is dominant and linear in the cell radius.

We propose a simple model in which motility forces in the bulk of a homogeneous monolayer are oriented by a dynamic pulling on cell–cell junctions. Our model combines conservation laws, monolayer mechanical properties, and a phenomenological coupling between strain and polarity: an advancing front cell pulls on its follower which becomes polarized after some time delay (possibly through the Merlin/Rac pathway), enabling signal propagation from the front back into the bulk; when the follower eventually increases its velocity, the front cell is free to increase its velocity too, generating time oscillations.

We find that waves appear spontaneously but are very close to the instability limit, which could explain why in the past waves were damped and difficult to extract from noise beyond one period. Parameter values close to the instability limit yield qualitative and quantitative predictions compatible with observations, including: waves propagate backwards; wave velocity increases with cell radius; lamellipodia inhibition attenuates, slows down or even suppresses the waves; cells maintain their polarity far from the migrating front. An interpretation of our results is that both cell and wave velocities depend on lamellipodia activity. This suggests that increasing traction forces, and/or decreasing the friction, would increase the cell and wave velocities; increasing the monolayer stiffness, and/or decreasing the friction, would increase the strain diffusion coefficient, and thus decrease the wave amplitude and frequency.

Together, our experiments and model disentangle the respective contributions of polarized active velocity and of proliferation in monolayer migration. They highlight the importance of coupling between non-autonomous strain on the one hand, and autonomous polarity and migration on the other, in collective cell migration and waves. They suggest that a cell on the substrate exerts a traction force which is cell-autonomous and linear in the cell radius, and that the ratio of cell stiffness to cell–substrate friction increases with cell radius. Finally, they reveal the central role of density in determining cell and wave velocities.

## Supplementary Material

Supporting Information

## Data accessibility

Figures supporting this article are provided in the electronic supplementary material. Movies, code and measurements supporting this article are available from the Dryad Digital Repository [74]: https://doi.org/10.5061/dryad.sk512.
