# Supplementary material for: Collective cell migration without proliferation: density determines cell velocity and wave velocity
Source: R Soc Open Sci. 2018 May 2;5(5):172421. doi: 10.1098/rsos.172421 (PMC5990758; doi:10.1098/rsos.172421)
Supplement: Supporting Information [file rsos172421supp1.pdf]

Collective cell migration without proliferation: density determines cell velocity and wave velocity

S. Tlili, E. Gauquelin, B. Li, O. Cardoso, B. Ladoux, H. Delanoë-Ayari, F. Graner

Supporting Information

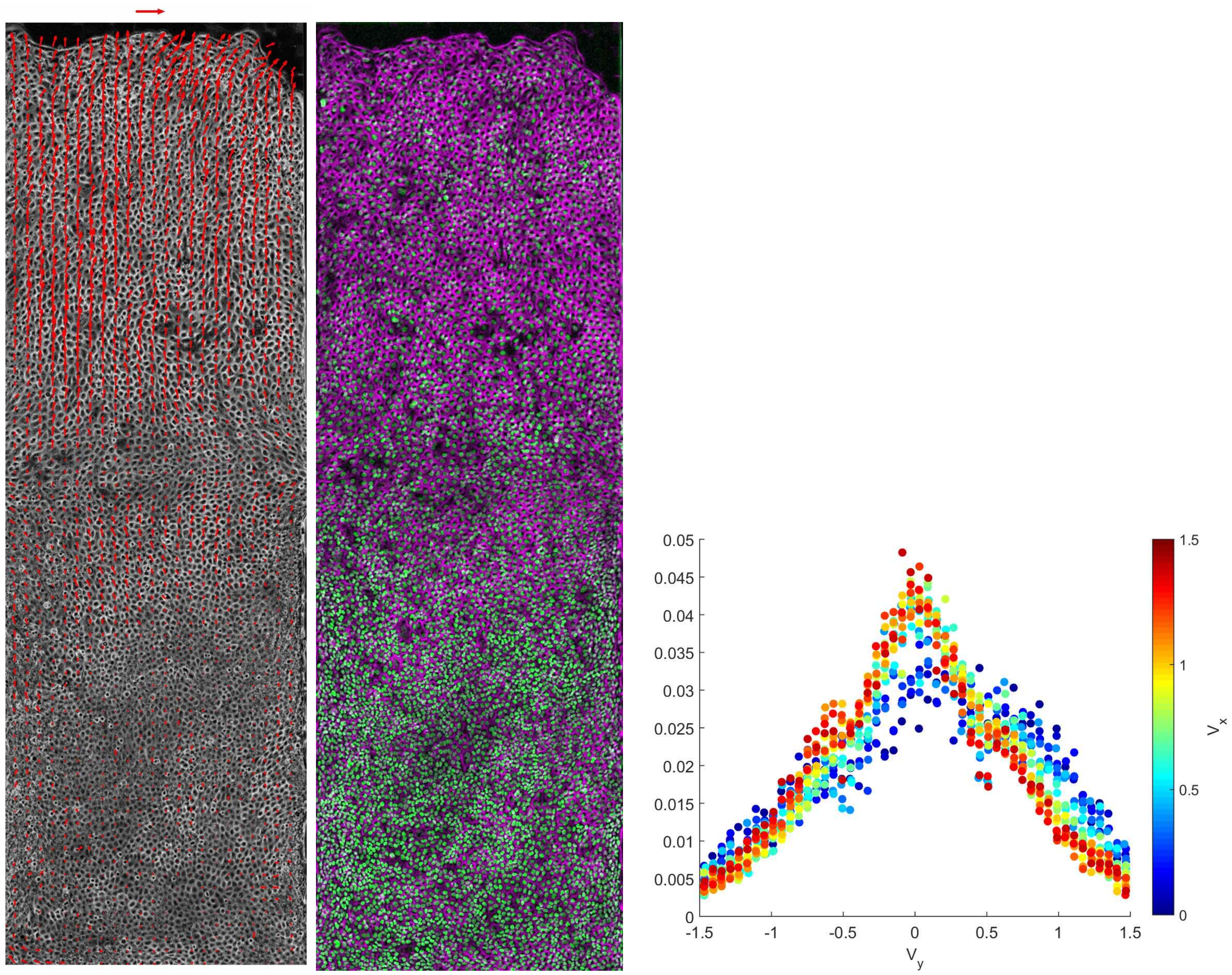

(A) (B) (C)

Supporting Figure S1. Cell velocity and density. *(A)* Instantaneous velocity field $\vec{v}(x, y, t)$ superimposed on cell contours, at $t = 15$ h after the first image (i.e. after around 20 h of migration). Scale arrow: 2 μm/min. Axis x from bottom to top. *(B)* Corresponding picture of nuclei labeled with histone GFP (green) evidence the large scale cell density variation; they can be detected for cell density measurement, and tracked for cell velocity validation. Cell contours appear in purple. *(C)* Distributions of y component of velocity field $\vec{v}(x, y, t)$, while its x component is indicated by the color code.

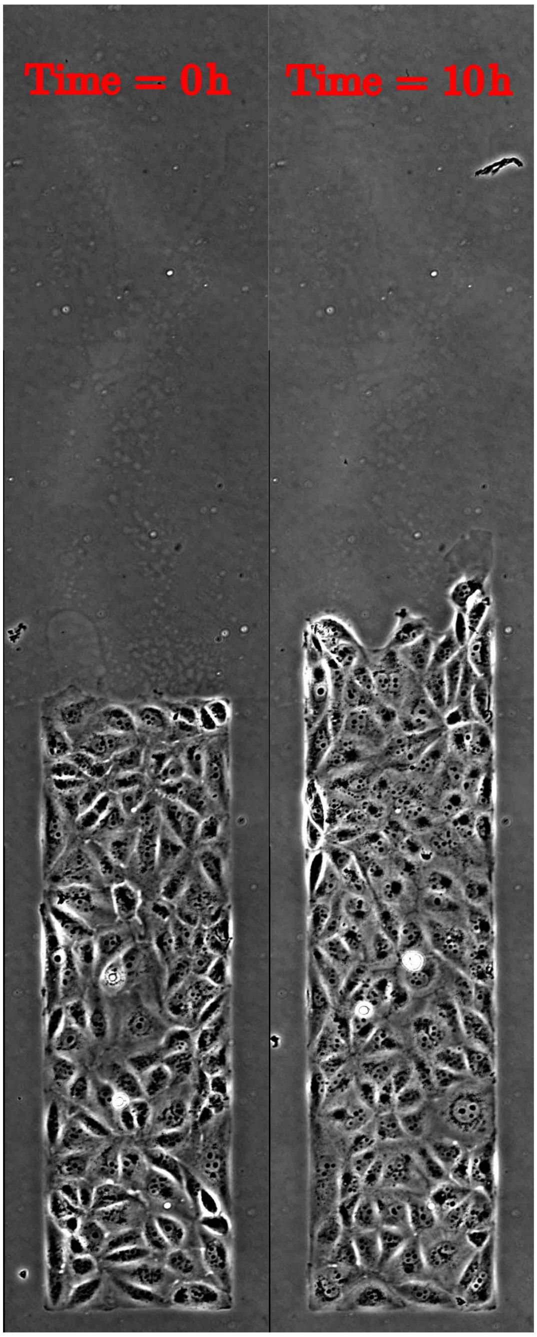

(A)

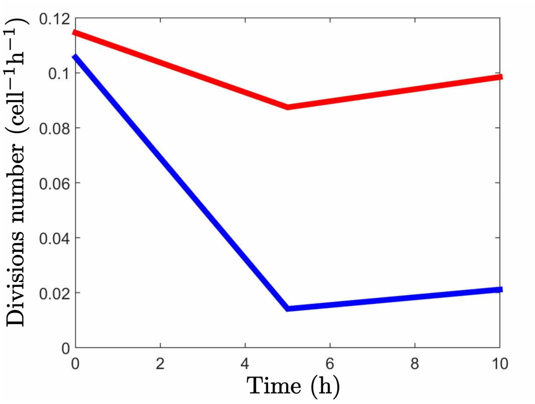

(B)

Supporting Figure S2. Effect of mitomycin. *(A)* First (left) and last (right) images used to test the effect of mitomycin during 10 hours after rinsing ($t = 0$). Strip width 200 μm. A small length is chosen, so that the whole experiment is visible on each image. Axis x from bottom to top. *(B)* Average division rate per cell and per hour. Blue: with mitomycin, rinsed at $t = 0$. Red: control, no mitomycin.

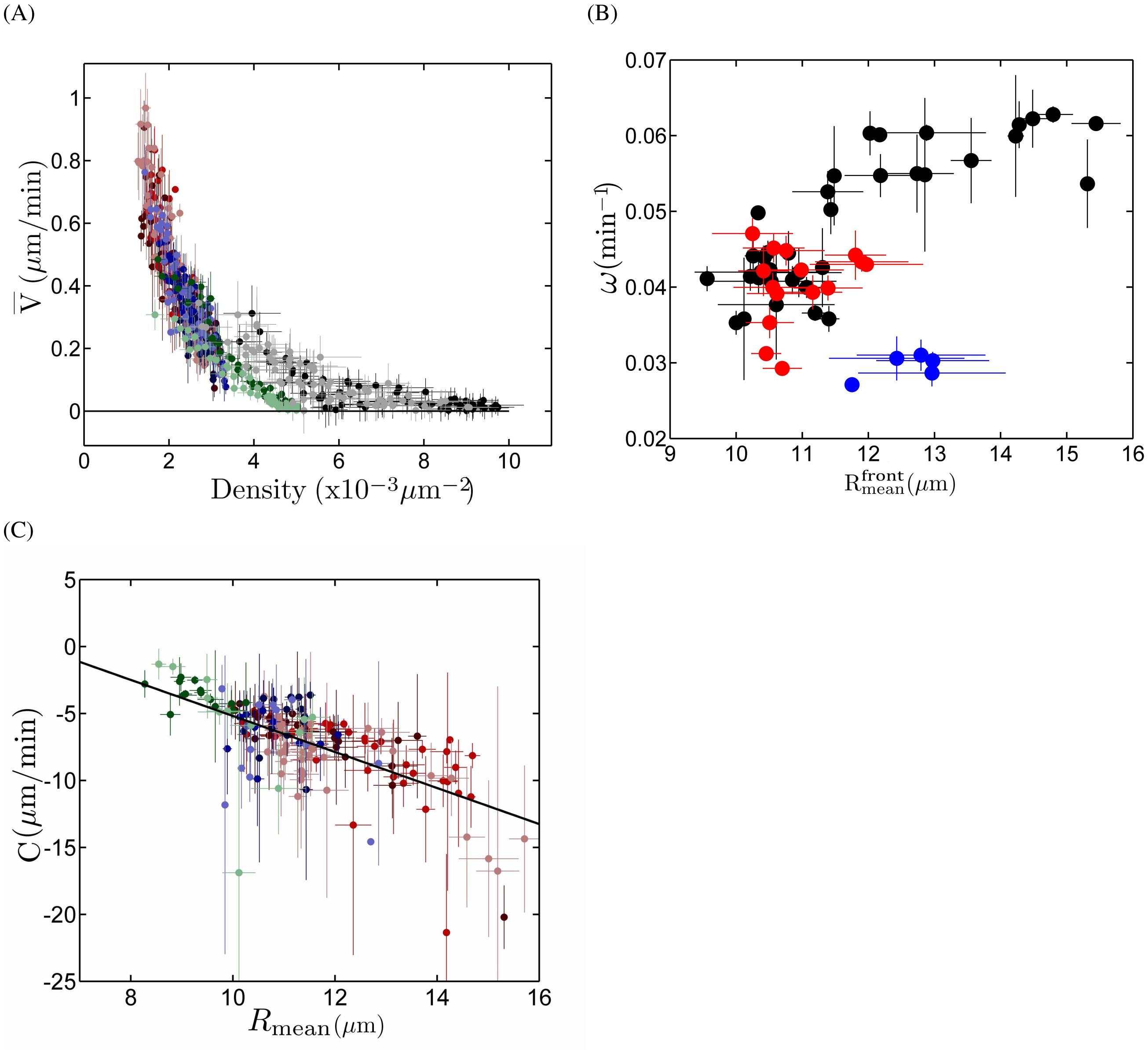

Supporting Figure S3. Variations with cell density. *(A)* Cell velocity $\bar{V}$ vs cell density $\bar{\rho}$. Same color code as in Fig. 2A,B. $N = 319$ with mitomycin C, colors; $N = 134$ in standard conditions, grey levels. *(B)* Angular frequency ω determined by wavelet analysis in the region of interest (which is manually selected according to its good signal-to-noise ratio, Fig. S5A), vs front value of mean effective cell radius R_{mean}: each point results from the spatial average $\pm$ SD both of ω and of R_{mean}, over a large spatial box close to the front (but excluding it), at a same time (binning 180 min $\times$ 528 μm). Black: with mitomycin C; experiments as in Fig. 3A, $N = 8$ strips. Red: experiments as in Fig. 4A, before adding CK666 drug; $N = 5$ strips. Blue, same after adding CK666 (points are measured at later times, and thus lower R_{mean}); $N = 3$ strips. Strips with signal-to-noise ratio too low to be measurable are not plotted. *(C)* Wave velocity c, determined by wavelet analysis in the same region of interest, versus mean effective cell radius R_{mean}, with a linear fit $c = 9.2 - 1.4\ R_{\mathrm{mean}}$ (R=0.59); $N = 155$ data points (one outlier has been removed).

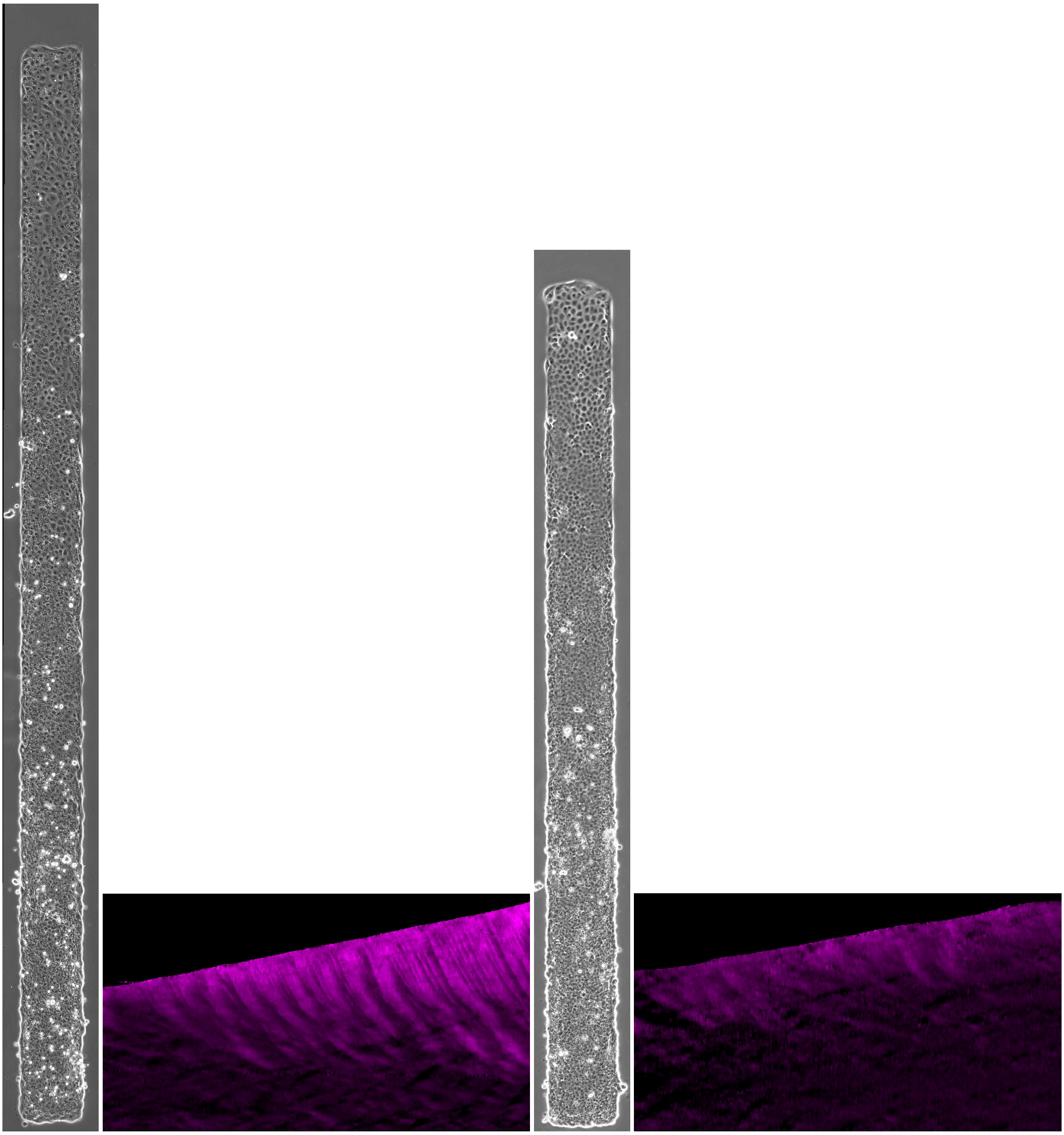

(A) (B) (C) (D)

Supporting Figure S4. Effect of divisions, in small width strips. *(A)* Experiment performed with mitomycin C to prevent divisions. Phase-contrast images showing cell contours. Axis x from bottom to top. *(B)* Corresponding kymograph of cell velocity $V(x,t)$ averaged over y but not over time. *(C,D)* Same under standard conditions (i.e. without mitomycin C). In *(A,C)*: Strip width 200 μm; migration from left to right; substrate: rigid PDMS. Despite the difference in the initial cell density, 10 10^{-3} μm^{-2} *(A)* vs 5 10^{-3} μm^{-2} *(C)*, at the time the picture is taken ($t = 30$ h) the density ranges in both experiments overlap. In *(B,D)*: Space x is from bottom (0 mm) to top (3.8 mm in B, 2.7 mm in D), time t from left (0 h) to right (30 h), and the top-left region is the bare substrate in front of the monolayer. Color code V from -0.15 (black) to 1.15 μm/min (purple).

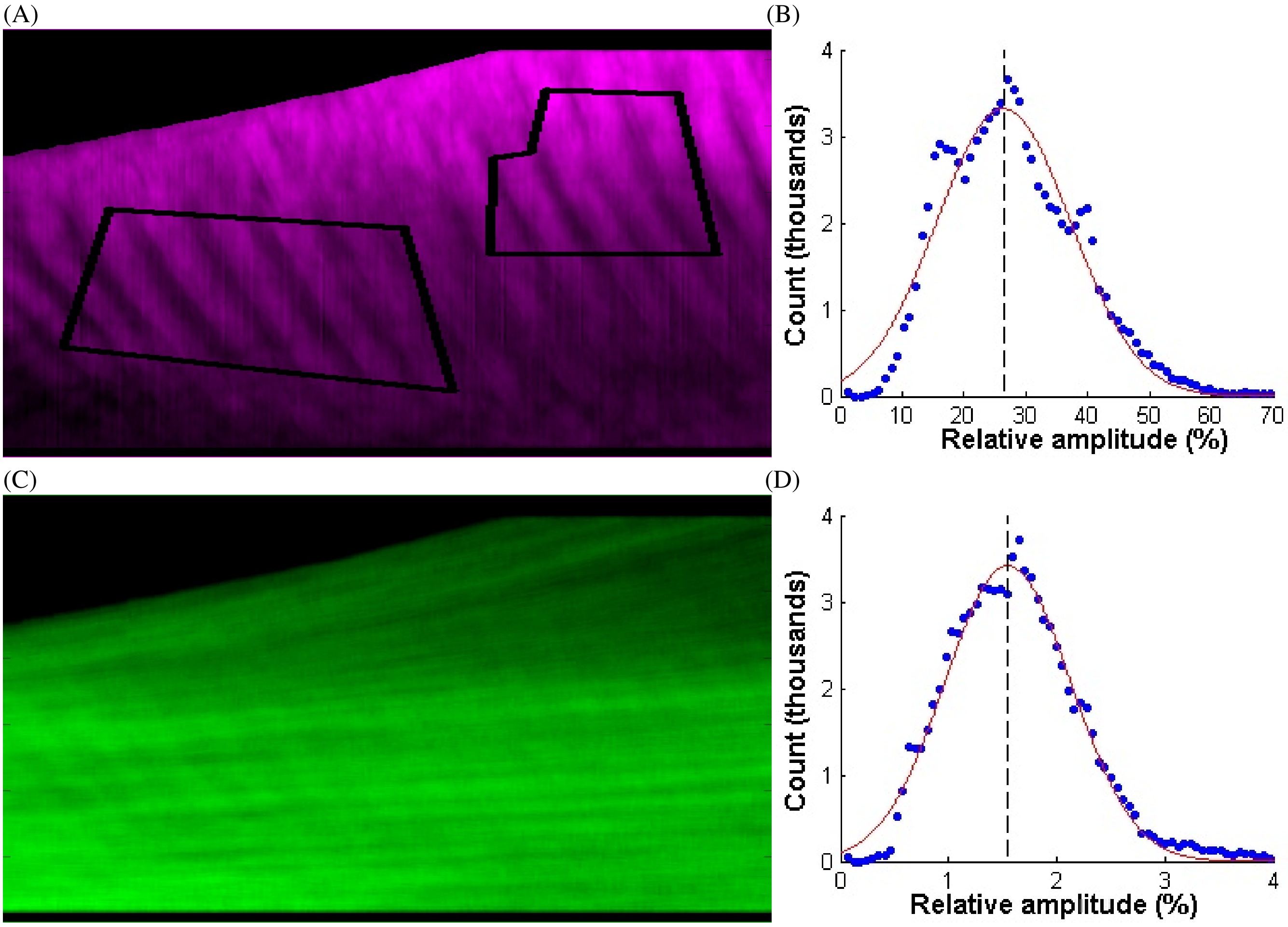

Supporting Figure S5. Local spatial variations. *(A)* Cell velocity $V(x,t)$ averaged over y but not over time. Space x is from bottom (0 mm) to top (3 mm), time t from left (0 h) to right (25 h), and the top-left region is the bare substrate in front of the monolayer. Color code $\bar{V}$ from -0.15 (black) to 1.15 μm/min (purple), $\bar{\rho}$ from 0.5 10^{-3} μm^{-2} (black) to 3.5 10^{-3} μm^{-2} (green). The outlined region of interest is selected manually as the region where waves have a good signal-to-noise ratio, large enough to perform the wavelet analysis. The results presented here are robust with respect to this manual selection. *(B)* Histogram of relative amplitude of wave velocity cumulated for 8 different experiments. Red line: Gaussian fit, of average 26.4% (marked by black dashes) and width 15.3%. *(C,D)* Same for density. Gaussian fit: average 1.55% and width 0.83%.

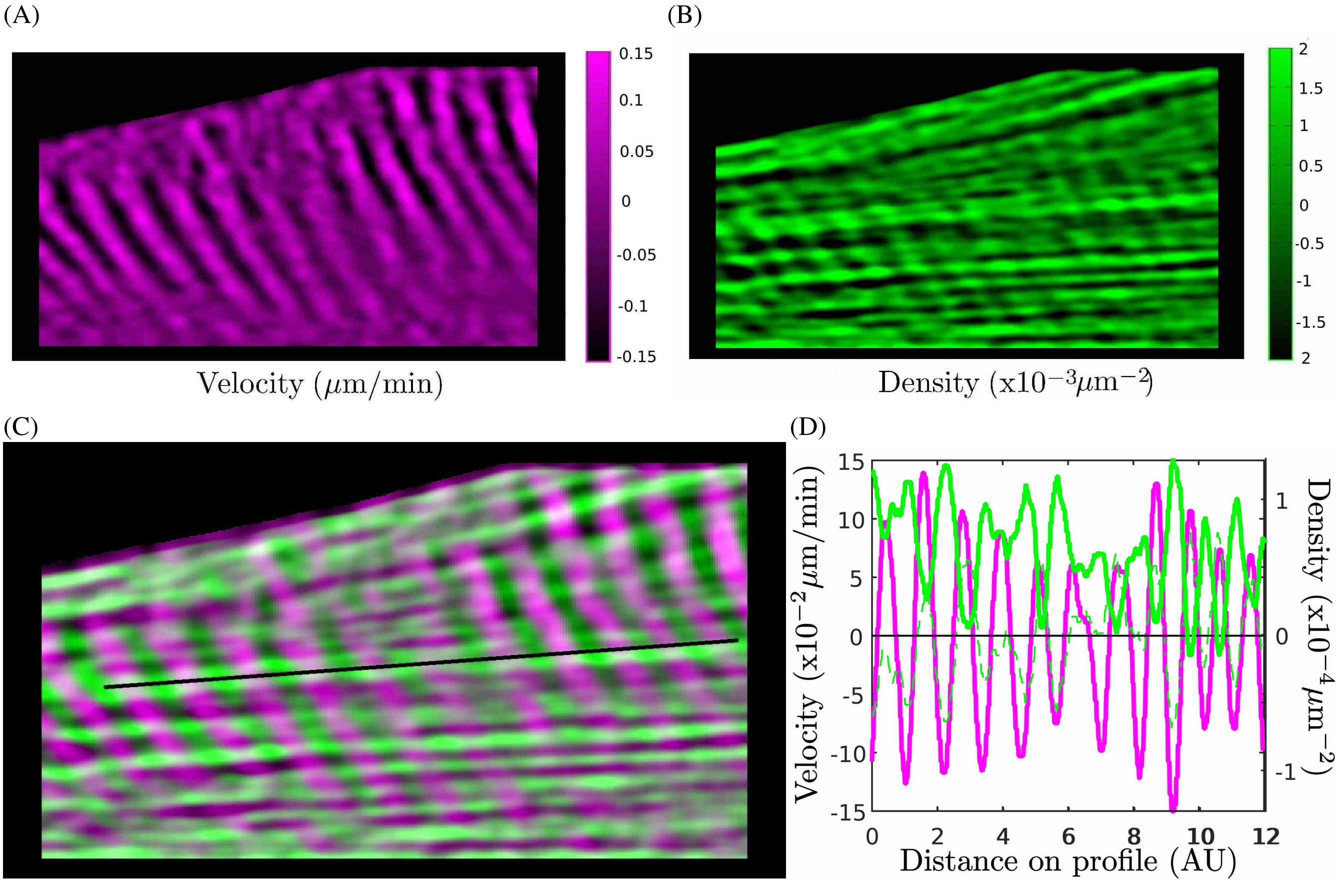

Supporting Figure S6.

Propagating waves. *(A-C)* Kymograph of small scale variations in *(A)* cell velocity, $\widetilde{V} = V - \bar{V}$, *(B)* in density, $\widetilde{\rho} = \rho - \bar{\rho}$, and *(C)* merge. Space x is from bottom (0 mm) to top (3 mm), time t from left (0 h) to right (25 h), and the top-left region is the bare substrate in front of the monolayer. Color code $\widetilde{V}$ from -0.15 μm/min (black) to 0.15 μm/min (purple), $\widetilde{\rho}$ from $-1.3\ 10^{-3}$ μm^{-2} (black) to $1.3\ 10^{-3}$ μm^{-2} (green). The line is manually drawn along a local density maximum. *(D)* Plot of $\widetilde{V}$ (purple) and $\widetilde{\rho}$ (green) along the line manually drawn in *(C)*. Inverting the sign of $\widetilde{\rho}$ (green dashes) evidences its phase opposition with $\widetilde{V}$, which agrees with Eq. (4.2).

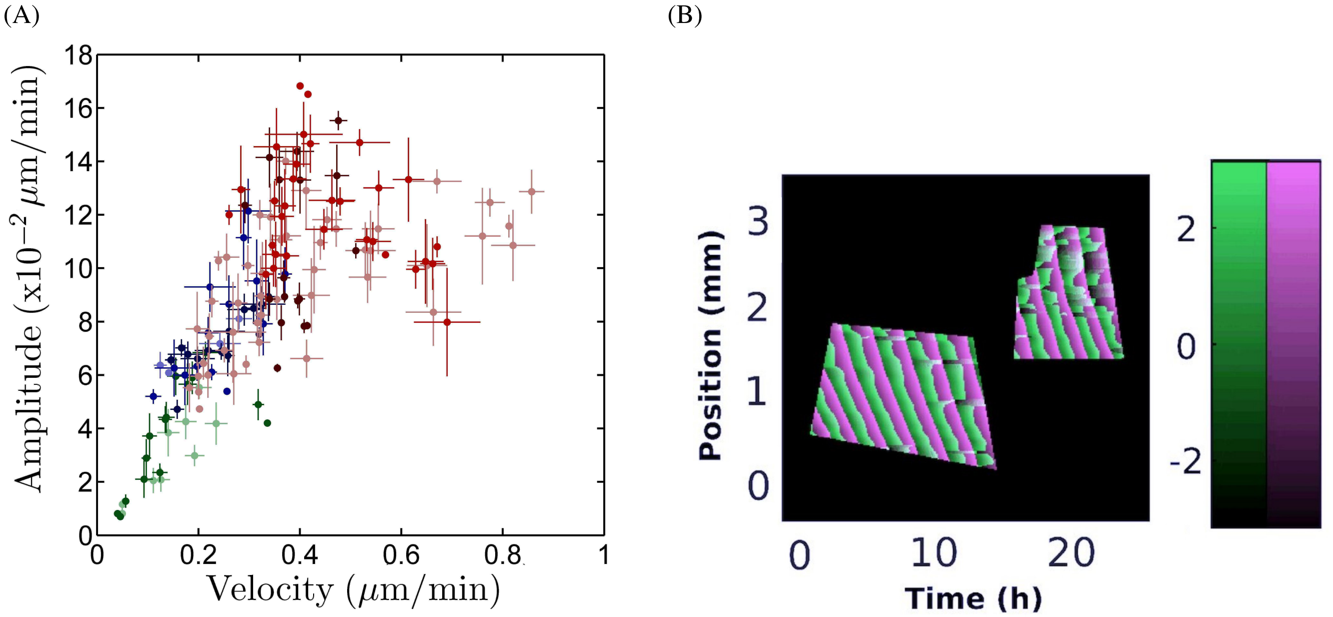

Supporting Figure S7. Local wave characteristics. They are determined by wavelet analysis in the region of interest, which is manually selected according to its good signal-to-noise ratio (Fig. S5A). *(A)* Amplitude $|\widetilde{V}|$ ($\pm$ SD) of velocity waves, versus average cell velocity $\bar{V}$; $N = 156$ data points. Same color code as in Fig. 2A,B. *(B)* Merged kymographs of phases: ϕ_ρ from $-\pi$ (black) to π (green), and ϕ_V from $-\pi$ (black) to π (purple). Space x is from bottom (0 mm) to top (3 mm), time t from left (0 h) to right (25 h).

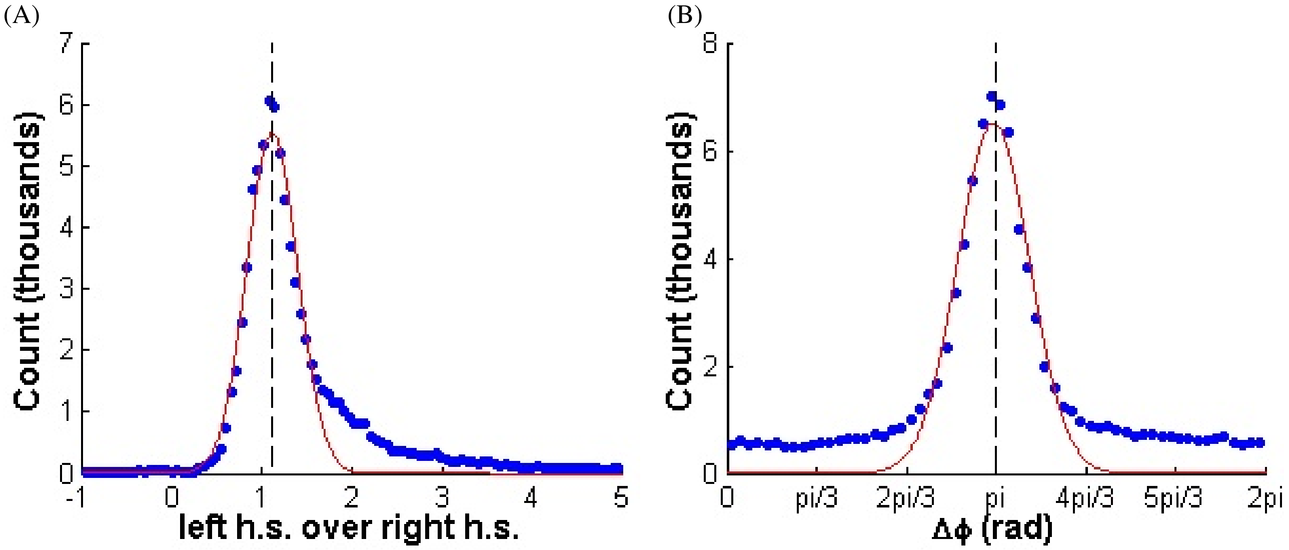

Supporting Figure S8. Test of equation Eq. (4.2) for density. *(A)* Histogram, cumulated for 8 different experiments, of the ratio of Eq. (4.2) left hand side (h.s.), $|\widetilde{\rho}|\left(|c|+\bar{V}\right)$ to right hand side, $\bar{\rho}|\widetilde{V}|$. Red line : Gaussian fit, of average 1.11 (vertical dashes). *(B)* Histogram of the difference $\phi_\rho - \phi_V$. Red line : Gaussian fit, of average 3.12 (vertical dashes).

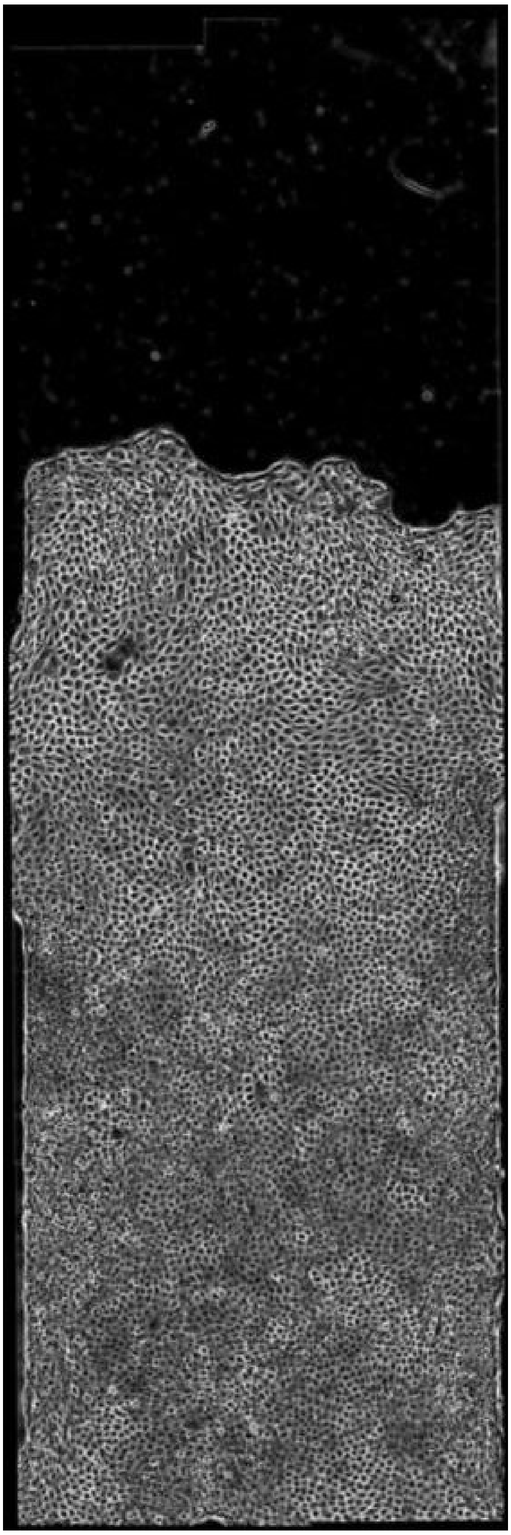

Supporting Movie S1. Monolayer of MDCK cells migrating within a straight strip with mitomycin C to prevent divisions. Phase-contrast image showing cell contours. Strip total length 4 mm, width 1 mm. Axis x from bottom to top. The first image, noted $t = 0$, is taken after around 5 h of migration. Duration of the movie: 26 h. To decrease the file size, the movie resolution has been decreased, and the time interval between frame has been doubled (10 min instead of 5 min in the original).

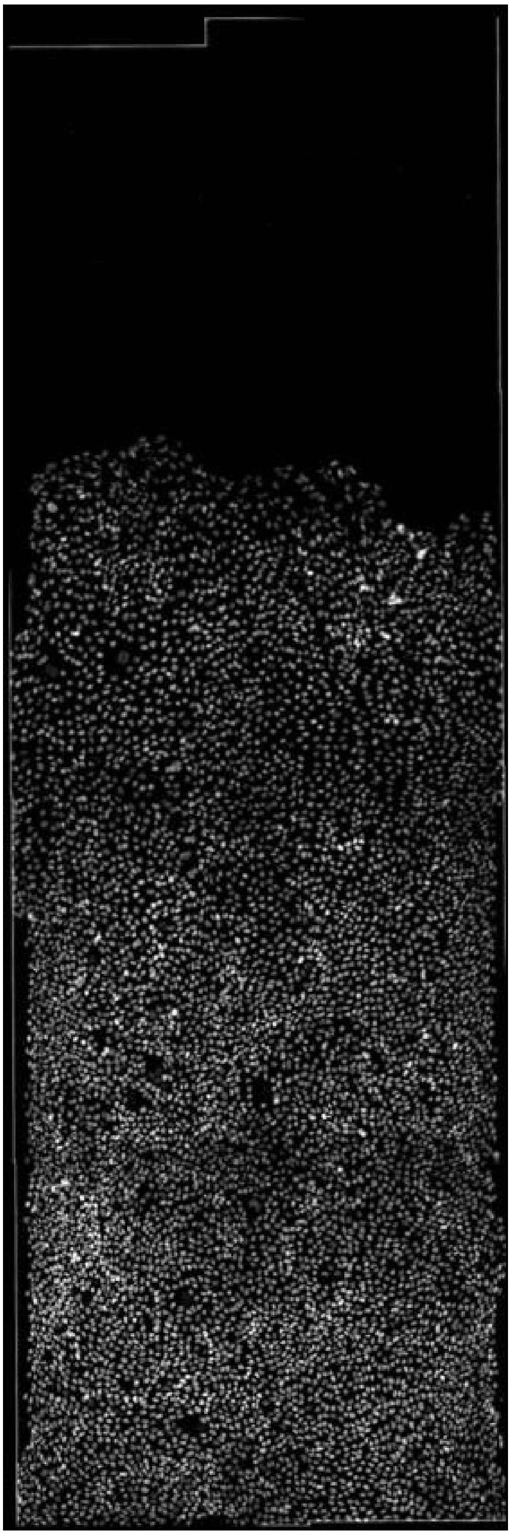

Supporting Movie S2. Same as Movie S1, nuclei labeled with histone GFP.

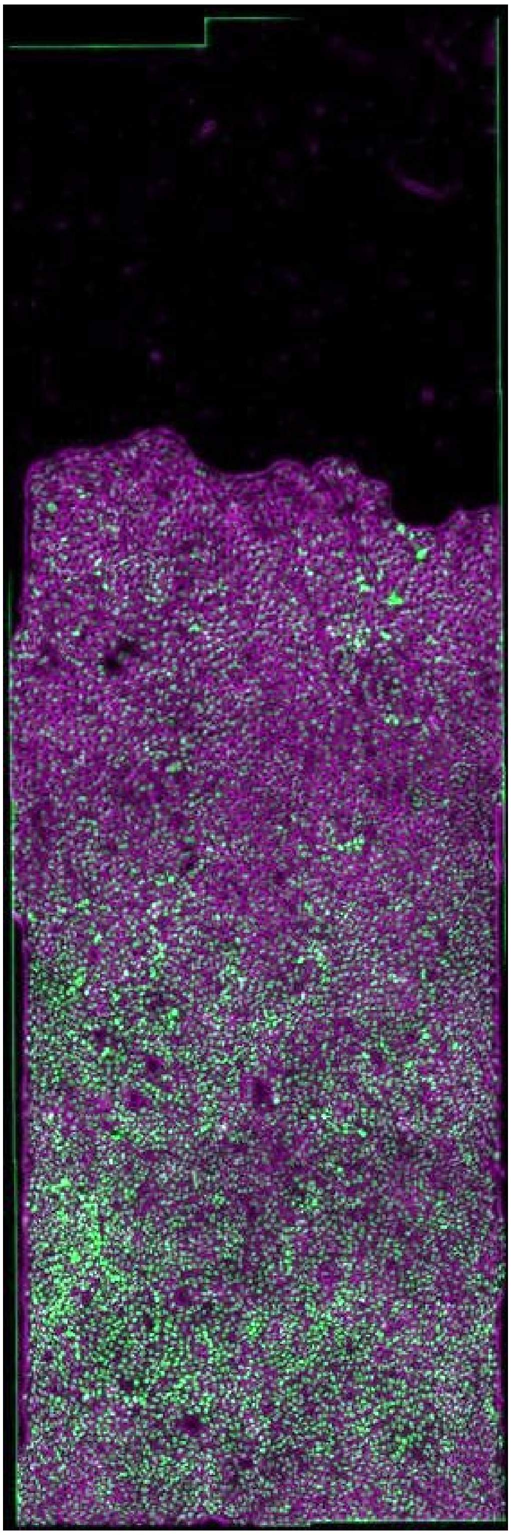

Supporting Movie S3. Merge of phase-contrast image showing cell contours (Movie S1) in purple, and nuclei labeled with histone GFP (Movie S2) in green.

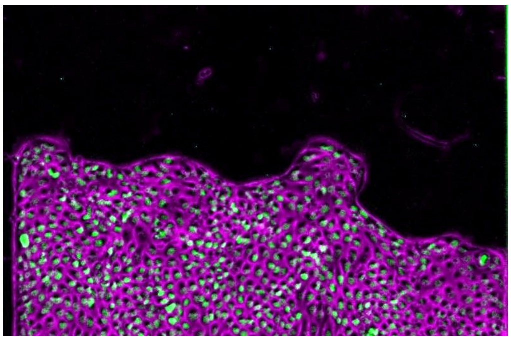

SI Supporting Movie S4. Same as Movie S3, with original time interval between frame (5 min), zoomed on the front.

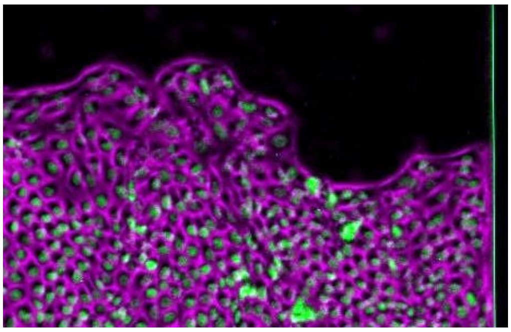

Supporting Movie S5. Same as Movie S3, with original time interval between frame (5 min), zoomed on the middle.

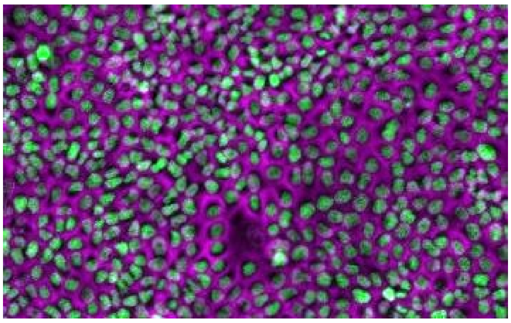

Supporting Movie S6. Same as Movie S3, with original time interval between frame (5 min), zoomed on the back.

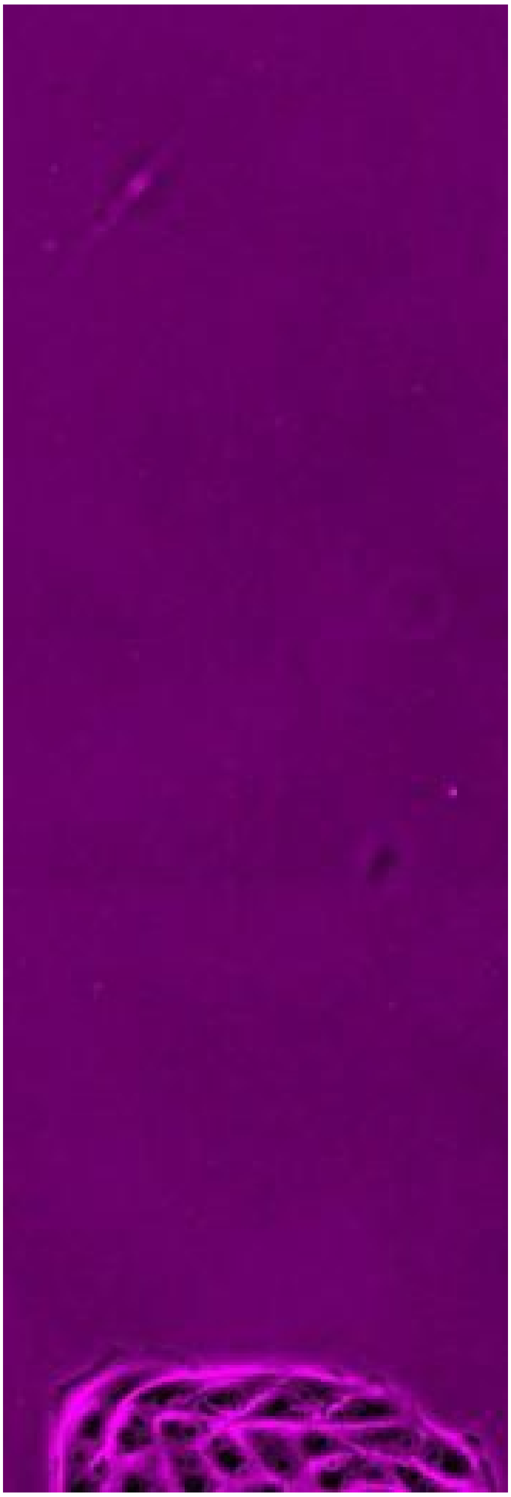

Supporting Movie S7. Same as Movie S3 (time interval between frames: 6 min). After 4 h (corresponding to the missing image at the 10th second of the current movie), CK666 drug is added; lamellipodia (both cryptic and front ones) are no longer detectable.
